# Challenges and Advances in Antemortem Diagnosis of Human Transmissible Spongiform Encephalopathies

**DOI:** 10.3389/fbioe.2020.585896

**Published:** 2020-10-20

**Authors:** Lucas M. Ascari, Stephanie C. Rocha, Priscila B. Gonçalves, Tuane C. R. G. Vieira, Yraima Cordeiro

**Affiliations:** ^1^Faculty of Pharmacy, Pharmaceutical Biotechnology Department, Federal University of Rio de Janeiro, Rio de Janeiro, Brazil; ^2^Institute of Medical Biochemistry Leopoldo de Meis, National Institute of Science and Technology for Structural Biology and Bioimaging, Federal University of Rio de Janeiro, Rio de Janeiro, Brazil

**Keywords:** prion diseases, prion protein, diagnostic, cell-free conversion assay, neurodegenerative disease

## Abstract

Transmissible spongiform encephalopathies (TSEs), also known as prion diseases, arise from the structural conversion of the monomeric, cellular prion protein (PrP^C^) into its multimeric scrapie form (PrP^Sc^). These pathologies comprise a group of intractable, rapidly evolving neurodegenerative diseases. Currently, a definitive diagnosis of TSE relies on the detection of PrP^Sc^ and/or the identification of pathognomonic histological features in brain tissue samples, which are usually obtained postmortem or, in rare cases, by brain biopsy (antemortem). Over the past two decades, several paraclinical tests for antemortem diagnosis have been developed to preclude the need for brain samples. Some of these alternative methods have been validated and can provide a probable diagnosis when combined with clinical evaluation. Paraclinical tests include *in vitro* cell-free conversion techniques, such as the real-time quaking-induced conversion (RT-QuIC), as well as immunoassays, electroencephalography (EEG), and brain bioimaging methods, such as magnetic resonance imaging (MRI), whose importance has increased over the years. PrP^Sc^ is the main biomarker in TSEs, and the RT-QuIC assay stands out for its ability to detect PrP^Sc^ in cerebrospinal fluid (CSF), olfactory mucosa, and dermatome skin samples with high sensitivity and specificity. Other biochemical biomarkers are the proteins 14-3-3, tau, neuron-specific enolase (NSE), astroglial protein S100B, α-synuclein, and neurofilament light chain protein (NFL), but they are not specific for TSEs. This paper reviews the techniques employed for definite diagnosis, as well as the clinical and paraclinical methods for possible and probable diagnosis, both those in use currently and those no longer employed. We also discuss current criteria, challenges, and perspectives for TSE diagnosis. An early and accurate diagnosis may allow earlier implementation of strategies to delay or stop disease progression.

## Prion Diseases

Prion diseases, also known as transmissible spongiform encephalopathies (TSEs), are a rare group of infectious, fatal neurodegenerative diseases caused by the deposition of misfolded prion protein particles in the brain (Prusiner, 1998; Scheckel and Aguzzi, 2018). The term “prion” was originally coined in 1982 by Stanley Prusiner to define the proteinaceous infectious particles that cause TSEs (Prusiner, 1982). The hallmark event in all TSEs is the conversion of the monomeric cellular prion protein (PrP^C^) into abnormally folded multimers, collectively termed prion scrapie (PrP^Sc^), which accumulate in the brain and display toxic and aggregation-prone properties (Prusiner, 1998; Requena and Wille, 2017; Baral et al., 2019).

These diseases progress slowly, with a long latency period prior to the manifestation of symptoms, which include ataxia, myoclonus, progressive dementia, depression, and general malaise. In contrast with other progressive neurodegenerative diseases, the period between the onset of symptoms and the TSE patient’s death is usually up to 1 year, making them rapidly progressive dementias (Prusiner and Hsiao, 1994; Chesebro, 2003; Wadsworth et al., 2003). Treatment for human TSEs remains symptomatic and supportive, as no cure is available to date (Geschwind, 2015; Forloni et al., 2019).

Transmissible spongiform encephalopathies are unique in medicine since they can have three origins: spontaneous (sporadic), genetic (familial), and acquired (infectious/transmitted). Human TSEs encompass diseases such as Kuru, Creutzfeldt–Jakob disease (CJD), Gerstmann–Sträussler–Scheinker syndrome (GSS), and fatal familial insomnia (FFI) (Zanusso et al., 2016; Ironside et al., 2017). Among human TSE cases, 80% to 95% are sporadic CJD (sCJD), 10% to 15% are genetic (often familial), and less than 1% are acquired (Geschwind, 2015). These diseases affect approximately 1–2 people per million worldwide annually (Chen and Dong, 2016). The human TSEs described above are summarized in Table 1.

**TABLE 1:** Transmissible spongiform encephalopathies (TSEs).

Disease	Form	Etiology	Clinical aspects	Incidence
Creutzfeldt–Jakob disease (CJD)	Sporadic (sCJD)	Either wild-type PrP^C^ converts spontaneously to PrP^Sc^, or a somatic mutation in the PrP gene (*PRNP*) renders PrP^C^ more susceptible to misfolding.	Rapidly progressive cognitive impairment with behavioral and visual disturbances, pyramidal and extrapyramidal signs, ataxia, and myoclonus.	The most common form of CJD (85%), with an annual incidence of 1.5 per million people. It generally occurs in late middle age (mean age of 67 years). Short survival post-diagnosis (about 4 months).
	Genetic (gCJD)	An inherited mutation in *PRNP* renders PrP^C^ more susceptible to misfolding.	Usually similar to sporadic CJD.	The frequency is estimated at 10%–15% of all forms of CJD. Those affected tend to be in their middle age when symptoms first arise.
	Iatrogenic (iCJD)	Human-to-human transmission occurs via cadaver-derived pituitary hormones, dura mater transplant, cornea transplant, neurosurgical instruments, or depth electrodes.	Usually similar to sporadic CJD.	Rare. The first case was reported in 1974 in a patient who had received a corneal transplant from a CJD-positive donor. Over 450 cases have been reported.
	Variant (vCJD)	Ingestion of meat from BSE-affected cattle provides exogenous PrP^Sc^ seed.	Psychiatric and sensory symptoms, ataxia, involuntary movements, and progressive cognitive impairment.	Rare. It was first reported in 1996 in the United Kingdom, where it reached epidemic proportions between the mid-1980s and 1996. In fact, most people who have developed vCJD have lived in the United Kingdom.
Fatal insomnia	Sporadic	Wild-type PrP^C^ converts spontaneously to PrP^Sc^ in patients lacking a genetic basis for this phenotype.	Cognitive decline, ataxia, psychiatric signs, and insomnia.	Rare. Twenty-five typical cases have been reported.
	Genetic (FFI)	Inherited D178N mutation coupled with the M129 genotype in *PRNP* renders PrP^C^ more susceptible to misfolding.	Insomnia, dysautonomia, ataxia, myoclonus, and epileptic seizures.	Rare. At least 70 families, 198 members of these families, and other 18 unrelated individuals are known to carry the D178N-129M allele (D178N mutation coupled with the M129 genotype).
Variably protease-sensitive prionopathy (VPSPr)	Sporadic	The abnormal PrP displays unusual biochemical properties. There are no mutations in *PRNP* and no risk factors for the development of acquired CJD.	Cognitive decline, psychiatric symptoms, and ataxia.	Rare. First described in 2008, with 11 cases identified in the United States.
Gerstmann–Sträussler–Scheinker syndrome (GSS)	Genetic	Inherited mutation in *PRNP* renders PrP^C^ more susceptible to misfolding. P102L is the most common mutation.	Slowly progressive ataxia with cognitive decline and parkinsonism later in the disease course.	Rare. First described in an Austrian family in 1936.
PrP systemic amyloidosis	Genetic	Premature termination of PrP caused by an inherent stop codon mutation renders PrP^C^ more susceptible to misfolding.	Sensory and/or sensorimotor autonomic neuropathy.	Rare. Reported in three families.

*References: Diack et al. (2014); Geschwind (2015); Zanusso et al. (2016); Ironside et al. (2017); Ritchie and Ironside (2017); Whitechurch et al. (2017); Cracco et al. (2018); Tee et al. (2018); Tesar et al. (2019); Uttley et al. (2020).*

Creutzfeldt–Jakob disease, the most common human prion disease worldwide, occurs mainly in the form of sporadic CJD (sCJD), which was first described in 1920 by German neurologist Hans Gerhard Creutzfeldt in a 23-year-old woman who had experienced fluctuating neuropsychological symptoms since adolescence. Shortly afterward, Alfons Maria Jakob described five other cases between 1921 and 1923, which he found to be similar to Creutzfeldt’s case (Iwasaki, 2017; Tee et al., 2018). There is no known genetic etiology of sCJD, nor are there known links between cases that are suggestive of an acquired disease; the assumption is, therefore, that cases of sCJD arise spontaneously (Ironside et al., 2017).

In 1987, a study described a prion disease in United Kingdom cattle named bovine spongiform encephalopathy (BSE) (Wells et al., 1987). Subsequently, in the 1990s, scientific and public attention was drawn to the appearance in the United Kingdom of a new human prion disease called variant CJD (vCJD), which was caused by the ingestion of meat from BSE-affected cattle (Will et al., 1996). In the late 1990s and early 2000s, some groups pointed to blood as a source of the scrapie agent, which at the time led to restrictions on blood transfusion in the United Kingdom as a way to prevent human-to-human transmission of vCJD (Turner and Ironside, 1998; Hunter et al., 2002; Llewelyn et al., 2004). A more recent study has shown that prion transmission in sheep via blood transfusion is indeed very efficient (Andréoletti et al., 2012).

Prior to the emergence of BSE, there were numerous reports of iatrogenic CJD (iCJD) cases in different countries in the 1970s and 1980s. These transmissions occurred via one of the following routes: treatment with human cadaver-derived growth hormone or gonadotropin, dura mater graft or corneal graft transplantation, and medical operations with neurosurgical instruments or depth electrodes (Will, 2003). Conventional sterilization protocols are ineffective in eradicating the PrP^Sc^ content in surgical instruments, and the methods recommended by the World Health Organization (WHO) are damaging to metal instruments and not practical for routine application (Thomas et al., 2013).

Despite the rarity of TSEs, these diseases represent a significant concern, not only because of their puzzling etiological aspects but also because of their considerable threat to public health. The development of non-invasive diagnostic methods for the early stages of TSEs is crucial in order to enable (*i*) the prevention of PrP^Sc^ spread, (*ii*) the implementation of potential therapies during earlier stages of the disease, and (*iii*) improvement in patient well-being. The development and validation of diagnostic methods for the detection at a preclinical stage are essential.

## The Prion Protein

Mature PrP^C^ is a 208-residue protein constitutively found on the surface of many cells, particularly neurons (Peggion et al., 2017). Before reaching the cell surface, a 253-residue precursor of PrP^C^ undergoes some post-translational modifications: the excision of a 22-residue segment at the N-terminus and a 23-residue segment at the C-terminus, the establishment of a disulfide bond between cysteine residues 179 and 214, and the attachment of a glycosylphosphatidylinositol (GPI) anchor to the C-terminus (Prusiner, 1991). Recent findings have suggested that PrP^C^ plays a central role in a variety of neurodegenerative diseases and could be a common therapeutic target for these disorders (Ayers and Prusiner, 2020; Corbett et al., 2020).

**FIGURE 1:**
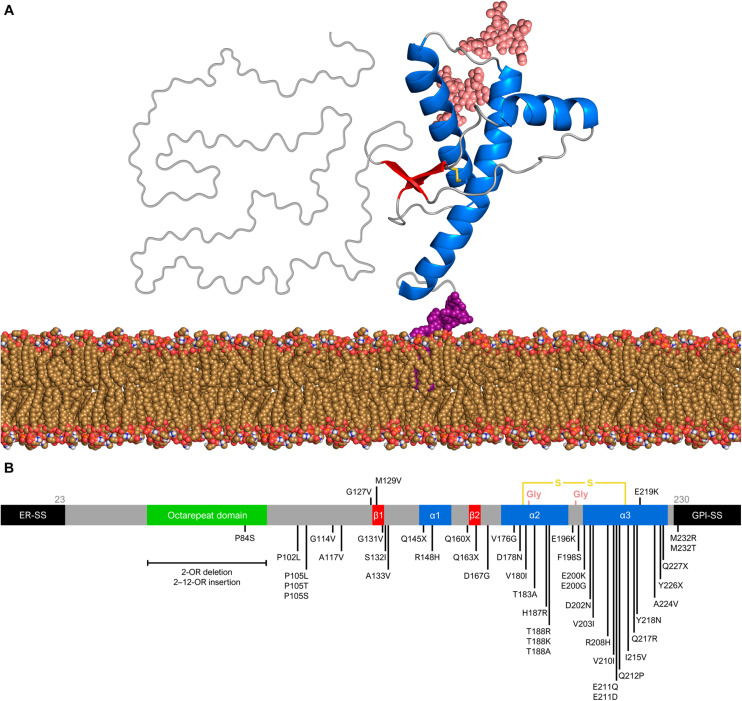
Prion protein structure and genetic variation. **(A)** Diglycosylated, full-length human PrP^C^ (huPrP^23–230^) attached to the membrane (yellow brown) by a GPI anchor (purple). The globular C-terminal domain comprises three α-helices (blue) and two small β-strands (red), while the N-terminal is intrinsically flexible. Turns and random coils are represented in gray, and the glycosides are shown in salmon. A disulfide bond (yellow) connects the α-helices 2 and 3. The globular domain structure was determined by nuclear magnetic resonance (PDB code 1QLX). **(B)** Schematic of human PrP primary structure, with disease-associated mutations (below) and polymorphisms (above). Deleterious mutations include missense and nonsense changes, as well as octarepeat insertions and deletions. The octarepeat domain (green) is a copper-binding segment of 4–5 contiguous repeats of the sequence PHGGGWGQ. The 22-residue endoplasmic reticulum signal sequence (ER-SS) and 23-residue GPI signal sequence (GPI-SS) at the ends are shown in black. The other portions are colored as in panel **(A)**.

The structure of PrP^C^ consists of an intrinsically unfolded N-terminal domain (residues 23–124) and a globular C-terminal domain (residues 125–230). The latter comprises three α-helices and a small, two-stranded β-sheet (Riek et al., 1996; Zahn et al., 2000; Requena and Wille, 2017) (Figure 1A). There are two glycosylation sites at asparagine residues 181 and 197, such that PrP^C^ exists in diglycosylated, monoglycosylated, and non-glycosylated forms (Prusiner, 1991). Despite its high conservation in mammals, the human PrP gene (*PRNP*) has over 40 deleterious mutations and a few polymorphisms (Figure 1B). The polymorphism at codon 129 (rs1799990) draws particular attention because it influences disease susceptibility and phenotype (Kim M. O. et al., 2018). Nearly 55% of the world population are homozygous for methionine (MM), while 9% are homozygous for valine (VV) and 36% are heterozygous (MV). The most common allele, M129, has a frequency of 97.5% in East Asians, 76% in South Asians, 67.5% in Europeans, 64.7% in Africans, and 59.4% in Hispanic Americans with Native ancestry (The 1000 Genomes Project Consortium, 2012).

**FIGURE 2:**
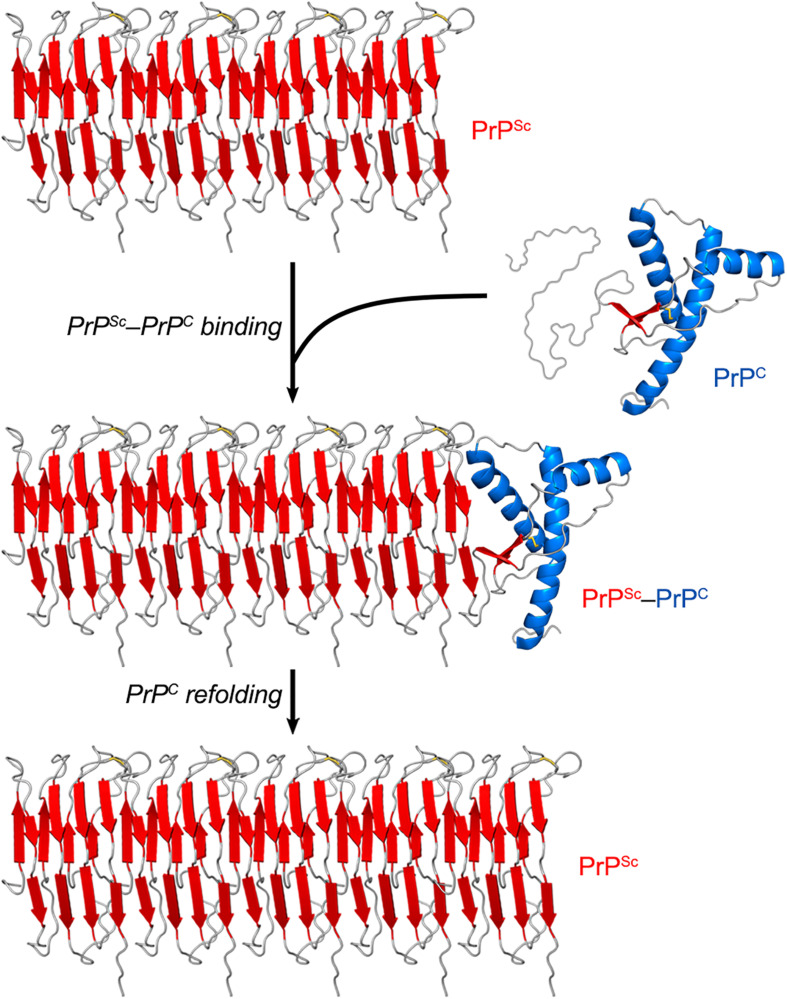
PrP^Sc^ amplification from PrP^C^. PrP^Sc^ particles grow by a nucleation process. A unit of PrP^C^ forms a complex with PrP^Sc^, and the latter induces the refolding of the former into a β-sheet-rich structure which then becomes part of the PrP^Sc^ multimer. In this self-propagating process, the PrP^Sc^ particles act like seeds/nuclei, with PrP^C^ serving as a substrate. The secondary structures are colored as follows: α-helices in blue, β-strands in red, and unstructured portions in gray. The atomistic model of PrP^Sc^ was constructed by combining experimental data and molecular dynamics simulations (Spagnolli et al., 2019).

While PrP^C^ has a predominantly α-helical structure, PrP^Sc^ consists of β-sheet-rich multimeric assemblies (Prusiner, 1998). Experimental evidence combined with computational approaches suggests that PrP^Sc^ consists of β-strands and relatively short turns and/or loops that form a β-solenoid architecture with a hydrophobic β-sheet core (Wasmer et al., 2008; Smirnovas et al., 2011; Spagnolli et al., 2019). A remarkable property of the PrP^Sc^ particles is that they are self-propagating—i.e., they act like seeds/nuclei and induce the refolding of PrP^C^ units, which are then integrated into PrP^Sc^ multimers. Thus, PrP^Sc^ particles grow by a nucleation process (Chiti and Dobson, 2006; Caughey et al., 2009; Cobb and Surewicz, 2009) (Figure 2). The pathogenic scrapie agent can spread from cell to cell and can transfer to a new host through the mechanisms outlined earlier (Acquatella-Tran Van Ba et al., 2013; Kraus et al., 2013).

PrP^Sc^ particles are not homogenous structures. Indeed, they can adopt different conformations. An intriguing consequence of this fact is that these distinct PrP^Sc^ species can be associated with different clinical characteristics of TSEs, including the latency period, brain damage pattern, and symptomatology. The concept of the prion strain has, therefore, been defined to represent this structural, biochemical, and neuropathological diversity (Aguzzi et al., 2007; Rossi et al., 2019). Some groups have even reported the artificial production of prion strains *in vitro*, and the infectivity and pathogenicity in animal models of these scrapie agents generated *de novo* (Colby and Prusiner, 2011; Kim C. et al., 2018). Notably, the diversity of PrP primary sequences and PrP^Sc^ conformations (strains) greatly affects the susceptibility of an organism to PrP^Sc^ from another organism, especially if these organisms belong to different species. It is thus common to talk about a species barrier or strain barrier for prion diseases (Scott et al., 2005; Sharma et al., 2016; Igel-Egalon et al., 2018).

A distinguishing feature between PrP^C^ and PrP^Sc^ is that the former has total protease sensitivity, whereas the latter generally has a protease-resistant core comprising the ∼90–230 region; hence, PrP^C^ and PrP^Sc^ are also referred to as sensitive PrP (^sen^PrP) and resistant PrP (^res^PrP), respectively (Prusiner, 1998; Caughey, 2000). Different strain-related PrP^Sc^ species can also have particular proteolytic digestion profiles, as they may differ in the length of their protease-resistant core (Bessen et al., 1995; Zou et al., 2003; Kobayashi et al., 2011; Kushnirov et al., 2020). Distinguishing between native PrP^C^ and infectious PrP^Sc^ based on their different biochemical properties is considered the “gold standard” approach for TSE diagnosis (Lukan et al., 2013; Haley and Richt, 2017). However, such an approach to discern different prion strains has yet to be exploited for diagnostic purposes.

## Diagnostic Approaches for Prion Diseases

At present, the definite diagnosis for TSEs is obtained via at least one of the following options: (*i*) identification of pathognomonic histological features in brain tissue sections, (*ii*) detection of ^res^PrP by immunohistochemical staining of brain tissue sections, or (*iii*) detection of ^res^PrP by immunoblotting of brain homogenate. Clinical evaluation and paraclinical tests only lead to a classification as possible or probable (Brown et al., 2003; CDC’s Diagnostic Criteria for Creutzfeldt–Jakob Disease (CJD), 2018). In the following sections, we will discuss the currently validated tests for definite, possible, and probable diagnosis, as well as other tests.

At this point, it is important to note two key terms in medical diagnosis: sensitivity and specificity. Sensitivity is the ability of a test to correctly identify true positive cases of the disease, whereas specificity is the ability of a test to identify true negative cases of the disease correctly. A high-sensitivity test produces more true positive results than false-negative results, while a high-specificity test produces more true negative results than false-positive results. The ultimate goal for diagnostic testing is to obtain sensitivity and specificity values as close to 100% as possible (Feinstein, 1975).

### Brain Tissue Examination

Transmissible spongiform encephalopathy patients’ brain tissue is characterized by vacuolization (spongiosis), neuronal death, dendritic and synaptic loss, astrogliosis, and ^res^PrP deposition. These features are most prominent in the cerebral and cerebellar cortexes. Vacuolization can be observed by histopathological examination. The vacuoles may, in fact, be an artifact generated by tissue processing (fixation, paraffin embedding, and staining). Apoptotic neurons can be identified by staining nuclei with DAPI (4′,6-diamidino-2-phenylindole) and/or immunochemical staining of caspase-3. Dendritic and synaptic loss can be revealed by Golgi silver staining. While astrogliosis is not specific to this group of diseases, it is consistently present and can be detected through immunochemical staining of glial fibrillary acidic protein (GFAP) (Aguzzi et al., 2001; Soto and Satani, 2011). However, these examinations have some drawbacks. Since tissue segments may have been collected from unaffected areas of the brain, these analyses offer limited diagnostic sensitivity and may well lead to false-negative results (Soto, 2004; Safar et al., 2005).

Immunohistochemical staining of brain tissue sections is a validated technique for detecting PrP^Sc^ in the brains of TSE-affected patients. To selectively stain for ^res^PrP^Sc^, brain tissue sections are previously subjected to limited proteolysis by proteinase K (PK) to eliminate the ^sen^PrP content (Bendheim et al., 1984; Parchi et al., 1999, 2012; Gambetti et al., 2003). This technique allows for higher diagnostic sensitivity than the brain tissue staining techniques mentioned above since it directly probes the etiological agent behind TSEs (Safar et al., 2005). However, the enzymatic pre-treatment does represent a significant drawback in terms of diagnostic sensitivity, since the PrP^Sc^ content may have varying degrees of resistance to proteolysis depending on the prion strain. Indeed, there have been reports of strain-associated PrP^Sc^ particles so poorly protease-resistant that they have been termed sensitive PrP^Sc^ (^sen^PrP^Sc^) (Safar et al., 2005; Pastrana et al., 2006; Sajnani et al., 2012). Limited proteolysis can thus result in digestion of a significant part of the whole PrP^Sc^ content, underestimating the total level of PrP^Sc^ in the brain (Safar et al., 2005).

One way to circumvent the limitations of the immunohistochemical examination is to skip the PK pre-treatment step and to perform conformation-dependent immunoassays (CDIs) using brain homogenate. CDIs markedly increase diagnostic sensitivity since they detect PrP^Sc^ species regardless of their protease resistance level (Safar et al., 1998, 2005; Kim et al., 2011, 2012). CDIs employ antibodies that recognize conformational epitopes rather than linear epitopes and are therefore selective for PrP^Sc^ over PrP^C^ (Kascsak et al., 1987; Bellon et al., 2003). Many conformational antibodies with selectivity for PrP^Sc^ have already been produced (Saijo et al., 2016). However, CDIs have not been tested in large cohorts and are thus not yet used in routine diagnostics or screening.

Another validated technique that is extensively used for diagnosis is Western blotting (immunoblotting) of brain homogenate, which also employs a pre-treatment with PK to digest the PrP^C^ content. This technique is particularly important because it provides information on several aspects of PrP^Sc^: its degree of proteolytic resistance, the size of the protease-resistant cores, and the ratio between the three different PrP glycoforms (un-, mono-, and diglycosylated). These features are correlated with the properties of individual strains as well as disease genotypes and phenotypes. The electrophoretic migration of the unglycosylated PK-resistant core is used to define two PrP^Sc^ types in cases of CJD: type 1, characterized by a ∼21-kDa band, and type 2, characterized by a ∼19-kDa band (Parchi et al., 1999). CJD subtypes are then categorized by codon 129 polymorphism and PrP^Sc^ type as MM1, MM2, MV1, MV2, VV1, and VV2. Type-1 PrP^Sc^ is mostly correlated with the 129 MM genotype, while type-2 PrP^Sc^ is generally correlated with the 129VV and 129MV genotypes; therefore, the most frequent subtypes are MM1, VV2, and MV2 (Parchi et al., 1999; Zerr et al., 2000b). With respect to PrP glycoforms, samples from sCJD patients predominantly contain the monoglycosylated band (Bendheim et al., 1984; Parchi et al., 1999, 2012; Gambetti et al., 2003). Meanwhile, FFI and vCJD are characterized by type-2 PrP^Sc^ and abundance of the diglycosylated band (Hill et al., 1997; Rossi et al., 1998).

Brain tissue examination is frequently performed postmortem, but brain biopsies provide an option for antemortem investigation. However, brain biopsies can lead to false-negative results since TSE pathology is variably distributed in the brain, and small samples may not contain pathognomonic histological features and/or detectable levels of PrP^Sc^. Because of their invasiveness, brain biopsies are very uncomfortable for the patients and can cause severe complications, including brain abscesses and hemorrhaging. Furthermore, the medical instruments used in neurosurgery must be appropriately discarded or subjected to WHO-recommended cleaning methods. Therefore, brain tissue examination is generally performed only postmortem (Brown et al., 2003).

### Neuropsychiatric Clinical Evaluation

The degeneration of certain areas of the brain, probably caused by PrP conversion and aggregation or by lack of functional PrP^C^, leads to the development of clinical signs that motivate patients to seek medical attention. Depending on their symptoms, a number of assessments of sCJD patients are possible. These include the cognitive or Heidenhain form of CJD, which accounts for the largest number of sCJD cases and is characterized by cognitive decline, cortical visual disturbances (involvement of the occipital cortex), mild psychiatric symptoms, and myoclonus. Cases can also be classified as ataxic or Brownell-Oppenheimer, a form that is characterized by cerebellar ataxia, cognitive decline, mood changes, and myoclonus at later stages, or as non-CJD phenotypes or Stern, characterized by presenile dementia, ataxia, and sleep disturbance (related to degeneration of the thalamus) (Puoti et al., 2012; Fragoso et al., 2017).

Gerstmann–Sträussler–Scheinker syndrome patients predominantly present cerebellar clinical features, progressive ataxia, incoordination, and late dementia. FFI patients’ main symptoms include sleep-related issues (insomnia, sleep-related dyspnea, sleep-related involuntary movements), and less frequently ataxia and psychiatric symptoms. Behavioral and psychiatric symptoms (excluding sleep disturbance) are also found in vCJD patients, along with persistent painful sensory symptoms, ataxia, myoclonus, and later dementia (Fragoso et al., 2017; Wu et al., 2018).

However, none of these signs alone are enough to diagnose prion diseases definitively. Subacute encephalopathy can be a consequence of infectious, inflammatory, autoimmune, or other causes, leading to diseases that should have their differential diagnosis because they are mostly treatable, unlike TSEs (Annus et al., 2016; Fragoso et al., 2017). For example, Hashimoto’s encephalopathy is a treatable disorder that results in cognitive decline, myoclonus, ataxia, and neuropsychiatric signs (Murray, 2011), similar to TSEs.

To determine a possible or probable TSE diagnosis, clinical symptoms must be consistent with paraclinical tests, which can include magnetic resonance imaging (MRI), electroencephalography (EEG), and CSF analysis (Soto, 2004; Araújo, 2013). However, this combination of clinical evaluation and paraclinical tests is insufficient for a definite TSE diagnosis, since biochemical and imaging alterations are not specific to the various TSE cases, as they are also present in other neurodegenerative conditions.

### *In vitro* Cell-Free PrP Conversion Assays

**FIGURE 3:**
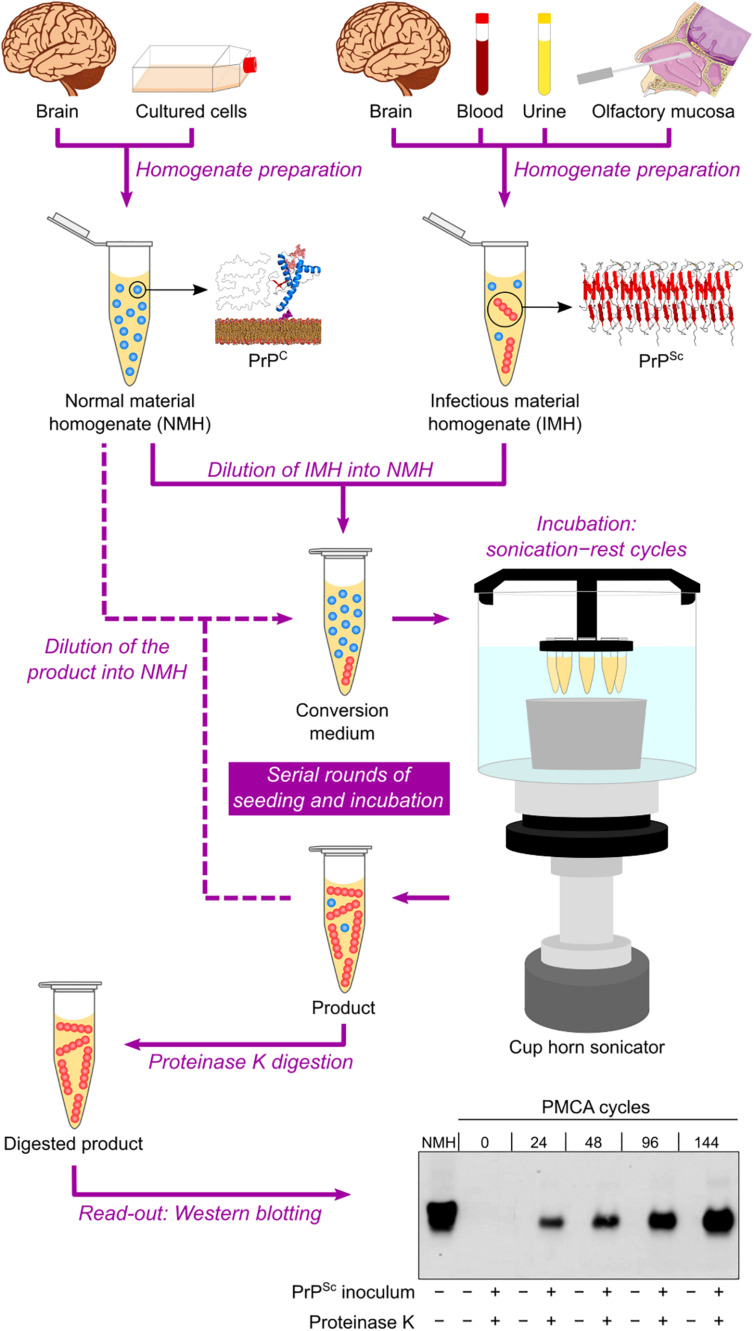
Protein misfolding cyclic amplification (PMCA) in automated fashion. Normal material homogenate (NMH), a source of PrP^C^ (substrate), is prepared from brain sections or cultured cells. Suspected infectious material homogenate (IMH), a possible source of PrP^Sc^ (seed), is prepared from brain sections, blood, urine, or olfactory mucosa brushings. Suspected IMH is diluted into NMH, and the resulting mixture is incubated with alternating cycles of sonication and rest. Subsequent rounds can be performed by diluting the product of the previous round into fresh NMH and repeating the incubation step. As a result, the amount of PrP^Sc^ is amplified after several sonication–rest cycles and even more after serial rounds of seeding and incubation. The products from different cycles and rounds are treated with proteinase K to eliminate PrP^C^ and then subjected to Western blotting to reveal the growing amount of ^res^PrP. This result indicates a positive TSE diagnosis. The Western blot shown is republished with permission of American Society for Biochemistry and Molecular Biology, from Ultra-efficient replication of infectious prions by automated protein misfolding cyclic amplification, Saá, Castilla, and Soto, 281, 46, 2006; permission conveyed through Copyright Clearance Center, Inc.

Based on the self-replicating ability of PrP^Sc^, a cell-free conversion assay called protein misfolding cyclic amplification (PMCA) was published at the beginning of the 2000s (Saborio et al., 2001). This technique consists of mixing PBS-diluted healthy brain homogenate and scrapie-infected brain homogenate (at a much higher dilution) in microtubes and then performing repeated cycles of incubation with orbital shaking at 37°C and sonication. Healthy and scrapie-infected brain homogenates are sources of PrP^C^/^sen^PrP (the reaction substrate) and PrP^Sc^/^res^PrP (the infectious seed), respectively. The incubation step allows aggregate growth, while the sonication step fragments these aggregates into smaller particles, which can act as seeds in the following incubation step. The reaction products are subjected to PK treatment and then to Western blotting to reveal their final ^res^PrP content. The result is an exponential increase in the amount of ^res^PrP per cycle (Saborio et al., 2001) (Figure 3).

The PMCA assay was later improved by optimizing brain homogenate preparation, medium composition, and sonication method, as well as by developing an automated option for the incubation–sonication step (Castilla et al., 2004; Saá et al., 2005) (Table 2 and Figure 3). These modifications allowed simultaneous runs of multiple samples with enhanced amplification efficiency (Castilla et al., 2005). The improved PMCA assay was shown to have very high sensitivity, as it significantly amplified as little as 10^–18^ g of PrP^Sc^ (equivalent to a 10^12^-fold dilution of SHB), as well as high specificity, as it never generated any detectable ^res^PrP in the absence of scrapie-infected brain homogenate inoculum, even after hundreds of PMCA cycles (Saá et al., 2006). When applied to human brain samples (collected either postmortem or by brain biopsy), PrP^Sc^ from sCJD and vCJD patients has been successfully amplified by PMCA (Soto et al., 2005; Jones et al., 2007; Oshita et al., 2016).

**TABLE 2:** *In vitro* cell-free PrP conversion assays.

Assay	Medium composition	Substrate	Container	Motion	Temperature	Sonication	Read-out
PMCA	Buffer system: H_2_PO_4_^–^, HPO_4_^2–^; Counterions: Na^+^, Cl^–^, K^+^; Chelating agent: EDTA; Surfactants: Triton X-100, (SDS); Protease inhibitors	PrP^C^ from brain or cell homogenate	Microtubes	Manual: orbital shaking (450 rpm); Automated: stationary	37°C	Manual or automated	PK treatment and Western blotting
rPrP-PMCA	Buffer system: H_2_PO_4_^–^, HPO_4_^2–^; Counterions: Na^+^, Cl^–^, K^+^; Surfactants: Triton X-100, SDS	Bacterially expressed, folded ^sen^rPrP	Microtubes	Stationary	37°C	Automated	PK treatment and Western blotting
ASA	Buffer system: H_2_PO_4_^–^, HPO_4_^2–^; Counterions: Na^+^, Cl^–^, K^+^; Chaotropic agent: guanidine; Fluorescent probe: ThT	Bacterially expressed, unfolded ^sen^rPrP	Multiwell microplate	Linear shaking	37°C	None	ThT fluorescence (real-time)
QuIC	Buffer system: H_2_PO_4_^–^, HPO_4_^2–^; Counterions: Na^+^, Cl^–^; Surfactants: Triton X-100, SDS	Bacterially expressed, folded ^sen^rPrP	Microtubes	Cycles of 1-min orbital shaking (1500 rpm) and 1-min rest	37–55°C	None	PK treatment and Western blotting
RT-QuIC	Buffer system: H_2_PO_4_^–^, HPO_4_^2–^; Counterions: Na^+^, Cl^–^; Chelating agent: EDTA; Surfactant: SDS; Fluorescent probe: ThT	Bacterially expressed, folded ^sen^rPrP	Multiwell microplate	Cycles of 1-min double orbital shaking (700 rpm) and 1-min rest	42–55°C	None	ThT fluorescence (real-time)

*ASA, amyloid seeding assay; EDTA, ethylenediaminetetraacetate; PK, proteinase K; PMCA, protein misfolding cyclic amplification; PrP^C^, cellular prion protein; QuiC, quaking-induced conversion; rPrP-PMCA, recombinant prion protein–protein misfolding cyclic amplification; RT-QuIC, real-time quaking-induced conversion; SDS, sodium dodecyl sulfate; ^sen^rPrP, sensitive recombinant prion protein; ThT, thioflavin T.*

A simpler and less expensive version of PMCA, called rPrP-PMCA, was developed by replacing PrP^C^ from brain or cell homogenate with bacterially expressed, folded recombinant ^sen^PrP (^sen^rPrP) (Table 2). The substrate concentration could thus be more accurately and efficiently defined in rPrP-PMCA than in classical PMCA (Atarashi et al., 2007). In the same year, another cell-free conversion technique, the amyloid seeding assay (ASA), was published (Colby et al., 2007). This cell-free conversion technique consists of loading a multiwell microplate with (*i*) guanidine-denatured ^sen^rPrP, (*ii*) PrP^Sc^ partially purified from scrapie-infected brain homogenate, (*iii*) guanidine, and (*iv*) thioflavin T (ThT). The latter component is a probe that emits fluorescence when it binds to amyloid structures. The mixture is incubated with continuous linear shaking at 37°C in a plate reader, which measures ThT fluorescence emission throughout the reaction, thus revealing the kinetics of aggregate formation (Colby et al., 2007) (Table 2).

Protein misfolding cyclic amplification (and its variation, rPrP-PMCA) and ASA have important differences. While it is challenging to deliver sound energy evenly to samples during sonication, lateral shaking subjects samples to the same motion, making ASA more practical and reproducible than the PMCA assays (Colby et al., 2007). The ASA technique is also faster and much more suitable for high-throughput investigations than PMCA since ASA relies on simple, automated, real-time, and fluorescence-based read-outs rather than time-consuming, immunoblotting-based read-outs (Colby et al., 2007). However, ASA has the disadvantage of frequently producing false-positive results, probably because of the presence of guanidine, a chaotropic agent, in the reaction medium (Colby et al., 2007).

A more refined cell-free conversion assay, the quaking-induced conversion (QuIC), was published more recently than the above techniques (Atarashi et al., 2008). The QuIC assay consists of loading microtubes with folded ^sen^rPrP and highly diluted scrapie-infected brain homogenate and then incubating the mixture at a controlled temperature with alternating cycles of orbital shaking and rest. The intermittent shaking results in separate aggregate fragmentation (seed formation) and aggregate growth phases, speeding up the conversion process. The read-out is performed by PK digestion and Western blotting, as in the PMCA assay (Table 2). The QuIC technique had high sensitivity, successfully amplifying as little as 10^–16^ g of PrP^Sc^ from brain homogenate, while also achieving high specificity, as it generated either no PK-resistant band or atypical, low molecular weight PK-resistant bands (Atarashi et al., 2008). The QuIC assay detected PrP^Sc^ with high sensitivity in brain homogenates from animals and humans and CSF samples from animals (Atarashi et al., 2008; Orrú et al., 2009).

**FIGURE 4:**
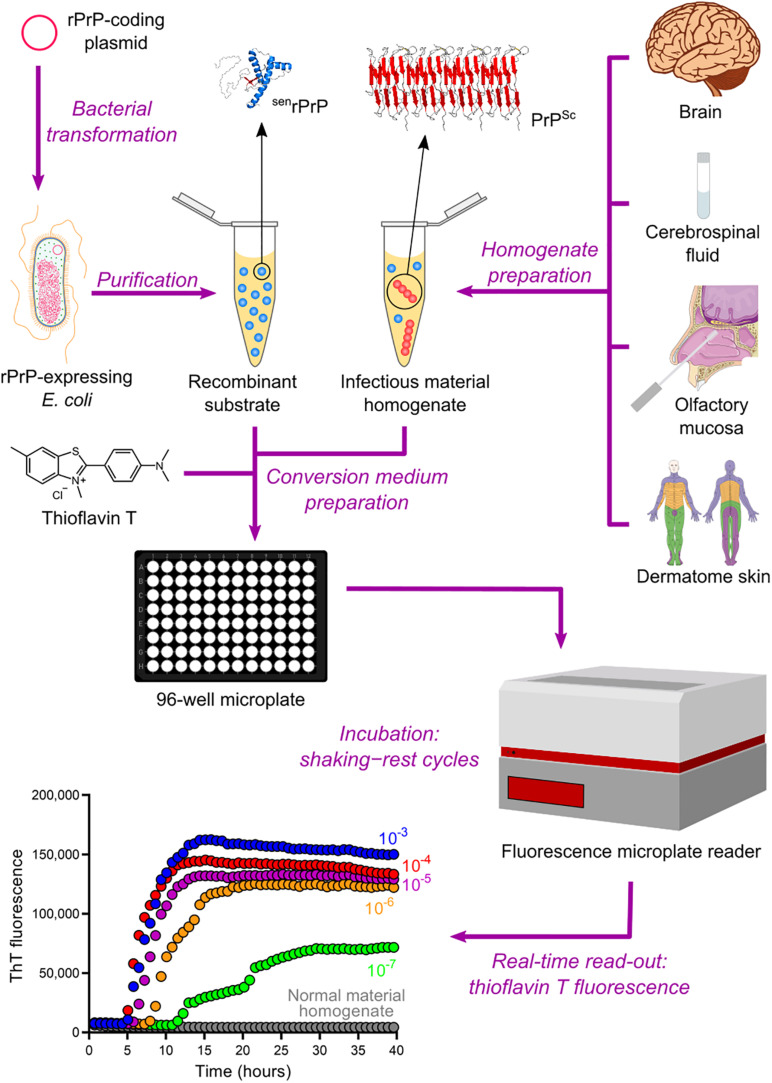
Real-time quaking-induced conversion (RT-QuIC). Recombinant PrP (rPrP) is expressed heterologously in *E. coli* and purified as folded protease-sensitive rPrP (^sen^rPrP), the substrate for RT-QuIC. Suspected infectious material homogenate, a possible source of PrP^Sc^ (seed), is prepared from brain sections, cerebrospinal fluid, olfactory mucosa brushings, and dermatome skin sections. High dilutions of a test sample are mixed with ^sen^rPrP, buffer, and thioflavin T (a fluorescent amyloid probe) to produce the conversion medium, which is loaded into a multiwell microplate and then incubated with alternating cycles of shaking and rest in a fluorescence microplate reader. This equipment records thioflavin T fluorescence emission throughout the incubation, thus revealing the kinetics of aggregate formation. A sigmoidal growth in thioflavin T fluorescence indicates a positive TSE diagnosis. The use of normal material homogenate does not lead to any increase in thioflavin T fluorescence, indicating that no conversion is happening. The positive result shown was obtained with brain homogenate from a patient with GSS.

The QuIC assay was then combined with the ASA read-out method to create the most sophisticated technique currently available for amplifying PrP^Sc^ *in vitro*, namely the real-time quaking-induced conversion (RT-QuIC) (Wilham et al., 2010). RT-QuIC differs from its predecessor in several steps. First, instead of microtubes, the conversion medium is supplemented with ThT and loaded into multiwell microplates. It is then incubated at 42°C with alternating cycles of double orbital shaking and rest and analyzed using real-time ThT fluorescence measurements rather than immunoblotting (Table 2 and Figure 4). The RT-QuIC technique is the fastest, most straightforward, most practical, least expensive, and most suitable cell-free conversion assay for high-throughput surveys. The RT-QuIC assay detected PrP^Sc^ at levels between ∼10^–15^ g and 10^–13^ g in brain homogenate, nasal fluids, and CSF from different animal species with high specificity (Wilham et al., 2010).

In the last decade, numerous studies have reported the detection of PrP^Sc^ in non-brain samples from human TSE patients by PMCA and especially RT-QuIC, as summarized in Table 3. The PMCA assay has been used to amplify PrP^Sc^ with high sensitivity and specificity from human samples such as blood (Lacroux et al., 2014; Bougard et al., 2016; Concha-Marambio et al., 2016) and urine (Moda et al., 2014). These studies used homogenates from the following biological materials as substrate sources: healthy human brain (Soto et al., 2005), human platelets (Jones et al., 2007), human PrP^C^-expressing transgenic mouse brain (Jones et al., 2007; Lacroux et al., 2014; Moda et al., 2014; Bougard et al., 2016; Concha-Marambio et al., 2016), and human PrP^C^-expressing 293F cells (Oshita et al., 2016). Another study has demonstrated that a CDI can be used as a read-out for PMCA to allow the detection of supposed ^sen^PrP^Sc^ species (Jones et al., 2007).

**TABLE 3:** Performance of PMCA and RT-QuIC for PrP^*Sc*^ detectionin non-brain samples from human patients with TSEs.

Study	Assay	Body Fluid	Sensitivity	Specificity
Atarashi et al., 2011	RT-QuIC	CSF	80% (sCJD)	100%
McGuire et al., 2012	RT-QuIC	CSF	87% (sCJD)	100%
Sano et al., 2013	RT-QuIC	CSF	83% (gCJD), 90% (GSS), 83% (FFI)	NP
Orrú et al., 2014	RT-QuIC	Olfactory mucosa	97% (sCJD), 100% (gCJD)	100%
	RT-QuIC	CSF	79% (sCJD), 50% (gCJD)	100%
Moda et al., 2014	PMCA	Urine	93% (vCJD)	100%
Orrú et al., 2015a	IQ-CSF	CSF	96% (sCJD)	100%
Cramm et al., 2016	RT-QuIC	CSF	80% (sCJD), 100% (gCJD), 57% (FFI)	99%
Bougard et al., 2016	PMCA	Plasma	100% (vCJD)	100%
McGuire et al., 2016	RT-QuIC	CSF	86%–100% (sCJD)	100%
Concha-Marambio et al., 2016	PMCA	Blood	100% (vCJD)	100%
Foutz et al., 2017	IQ-CSF	CSF	92%–95% (sCJD), 93%–100% (gCJD)	99–100%
Bongianni et al., 2017	RT-QuIC	Olfactory mucosa	97% (sCJD), 75% (gCJD/GSS)	100%
	RT-QuIC	CSF	72% (sCJD), 57% (gCJD/GSS)	NP
	IQ-CSF	CSF	86% (sCJD), 50% (gCJD)	100%
	RT-QuIC + IQ-CSF	CSF	95% (sCJD), 71% (gCJD/GSS)	100%
	RT-QuIC + IQ-CSF	Olfactory mucosa + CSF	100% (sCJD), 75% (gCJD/GSS)	100%
Groveman et al., 2016	RT-QuIC	CSF	73% (sCJD)	100%
	IQ-CSF	CSF	94% (sCJD)	100%
Franceschini et al., 2017	IQ-CSF	CSF	92% (sCJD), 100% (gCJD), 100% (iCJD), 25% (vCJD), 33% (GSS), 0% (FFI), 100% (VPSPr)	100%
Redaelli et al., 2017	PMCA	Olfactory mucosa	100% (FFI)	100%
	RT-QuIC	Olfactory mucosa	100% (FFI/sCJD/iCJD)	100%
Orrú et al., 2017	RT-QuIC	Dermatome skin	88%–94% (sCJD)	100%
Rudge et al., 2018	RT-QuIC	CSF	89% (sCJD)	100%
Hermann et al., 2018	RT-QuIC	CSF	89% (sCJD)	100%
Abu-Rumeileh et al., 2019	RT-QuIC	CSF	83% (sCJD), 86% (gCJD)	100%
	IQ-CSF	CSF	97% (sCJD), 100% (gCJD)	100%
Metrick et al., 2019	RT-QuIC	Olfactory mucosa	100% (sCJD)	100%
Fiorini et al., 2020	RT-QuIC	Olfactory mucosa	91% (sCJD)	100%
	IQ-CSF	CSF	96% (sCJD)	100%
	RT-QuIC + IQ-CSF	Olfactory mucosa + CSF	100% (sCJD)	100%

*Abbreviations: CJD, Creutzfeldt–Jakob disease; CSF, cerebrospinal fluid; FFI, fatal familial insomnia; gCJD, genetic Creutzfeldt–Jakob disease;GSS, Gerstmann–Sträussler–Scheinker syndrome; iCJD, iatrogenic Creutzfeldt–Jakob disease; IQ-CSF, improved RT-QuIC for CSF (2^*nd*^ generation); NP, not performed; PMCA, protein misfolding cyclic amplification; RT-QuIC, real-time quaking-induced conversion; sCJD, sporadic Creutzfeldt–Jakob disease; vCJD, variant Creutzfeldt–Jakob disease.*

Several studies have reported the high-sensitivity and high-specificity amplification of ^res^PrP using RT-QuIC with different human samples, including brain homogenate (Atarashi et al., 2011; Peden et al., 2012), CSF (Atarashi et al., 2011; McGuire et al., 2012, 2016; Sano et al., 2013; Orrú et al., 2014; Cramm et al., 2015, 2016; Hermann et al., 2018; Rudge et al., 2018), olfactory mucosa (Orrú et al., 2014; Bongianni et al., 2017; Fiorini et al., 2020), and dermatome skin (Orrú et al., 2017). Another study coupled immunoprecipitation of PrP^Sc^ from human plasma with RT-QuIC, thus avoiding interference from plasma components and increasing sensitivity up to 10^–18^ g of PrP^Sc^ (Orrú et al., 2011).

Since it is common practice to collect CSF samples from patients with suspected CJD in order to rule out other diseases, this bodily fluid is highly valuable for diagnosis (Chitravas et al., 2011). A second-generation RT-QuIC assay (with small modifications in the protocol) has been developed specifically to enhance sensitivity in CSF analyses and shorten reaction times (Orrú et al., 2015a). It was designated as improved RT-QuIC for CSF (IQ-CSF). Subsequent work achieved high-sensitivity diagnosis from human CSF samples by using IQ-CSF (Groveman et al., 2016; Bongianni et al., 2017; Foutz et al., 2017; Franceschini et al., 2017; Abu-Rumeileh et al., 2019; Fiorini et al., 2020).

Some studies have shown that bank voles (BV) have a uniquely weak species/strain barrier for prion transmission, as they are susceptible to multiple PrP^Sc^ strains from a wide range of species, including humans, sheep, deer, and hamsters (Nonno et al., 2006; Agrimi et al., 2008; Di Bari et al., 2008, 2013). Another study has shown that BV PrP^C^-expressing mice are susceptible to many prion strains from eight different species, including humans (Watts et al., 2014). Collectively, these data indicated that BV PrP^C^ was a universal substrate for conversion into PrP^Sc^, thus circumventing the need for suitable ^sen^PrP substrates (with different sequences) to identify distinct prion types using RT-QuIC and PMCA.

RT-QuIC reactions using BV ^sen^rPrP as substrate have been able to detect multiple human prion strains with high sensitivity in human samples including brain homogenate (Orrú et al., 2015b), olfactory mucosa (Redaelli et al., 2017), and dermatome skin samples (Orrú et al., 2017). These studies observed the generation of BV ^res^PrP species with different PK-resistance profiles, though without any clear correlation between the proteolytic digestion profiles and the different strains used as seed. Similarly, PMCA reactions using BV brain homogenate successfully amplified PrP^Sc^ from human olfactory mucosa samples, although the original glycoform ratio and the electrophoretic migration of the unglycosylated PK-resistant core of prion strains were not maintained (Redaelli et al., 2017). Even though the use of BV ^sen^PrP cannot provide valuable information on prion strains, this substrate does simplify and lower the cost of routine application of RT-QuIC and PMCA for TSE diagnosis.

An important study has reported the detection of PrP^Sc^ in skin samples of prion-infected rodents by PMCA and RT-QuIC at a preclinical stage of disease. Hamsters infected with 263K scrapie strain, which displayed clinical signs from 10 weeks post inoculation (wpi), were positively diagnosed at 2 wpi by PMCA and 3 wpi by RT-QuIC. Human PrP^C^-expressing mice infected with sCJD agent, which showed clinical signs at 24 wpi, were positively diagnosed at 4 wpi by PMCA and 20 wpi by RT-QuIC (Wang et al., 2019). These findings additionally highlight the enormous potential of *in vitro* cell-free PrP conversion assays for an early TSE diagnosis.

Protein misfolding cyclic amplification and RT-QuIC have important differences in terms of their end products. When PMCA products were inoculated intraperitoneally in wild-type hamsters, these animals developed the same disease pattern as hamsters inoculated with infectious brain material did, demonstrating that infectivity could also be amplified by PMCA (Saá et al., 2006). Conversely, when RT-QuIC products were inoculated intracerebrally in wild-type hamsters, their brains showed very low seeding activity but no histopathological changes, while Western blotting detected no ^res^PrP. This result indicated that RT-QuIC-generated ^res^PrP was neither infectious nor pathogenic, suggesting that RT-QuIC is much less biohazardous than PMCA for routine use (Groveman et al., 2017). In another study, sCJD-seeded RT-QuIC products inoculated intracerebrally in mice overexpressing human PrP^C^ elicited neither clinical signs of disease nor astrogliosis (Raymond et al., 2020). In fact, PMCA products are more similar to brain-derived PrP^Sc^, while the ^res^PrP species produced by RT-QuIC have shorter PK-resistant cores (McGuire et al., 2012; Groveman et al., 2015, 2017; Kraus et al., 2015), which may explain why PMCA and RT-QuIC end products are distinct in terms of infectivity. Thus, the methodological simplicity and biosafety of RT-QuIC over PMCA is probably the reason why RT-QuIC was the most employed assay for TSE diagnosis in robust studies published in the 2010s (Table 3).

A recent study has shown that the replacement of chloride anions with less hydrated bromide anions in the RT-QuIC medium significantly increased sensitivity and specificity for PrP^Sc^ amplification in brain and olfactory mucosa samples from sCJD patients (Metrick et al., 2019). This important finding may enable the RT-QuIC technique to be even more refined and distinguished in the years to come.

The RT-QuIC assay was first offered clinically in 2015 in the United States and became part of the Centers for Disease Control and Prevention (CDC) criteria in 2018. Currently, RT-QuIC, in combination with other techniques, characterizes TSE diagnosis only as probable, not definitive (CDC’s Diagnostic Criteria for Creutzfeldt–Jakob Disease (CJD), 2018). Outstandingly, two studies have reported sCJD diagnosis with 100% sensitivity and 100% specificity by combining RT-QuIC analysis of olfactory mucosa samples and IQ-CSF (Bongianni et al., 2017; Fiorini et al., 2020). Two other studies have shown that the association of IQ-CSF with other techniques—such as MRI, EEG, and 14-3-3 detection (discussed below)—resulted in sCJD diagnosis with at least 97% sensitivity and 99% specificity (Hermann et al., 2018; Rudge et al., 2018). Therefore, it is very plausible that a definitive antemortem diagnosis can be obtained by the combination of RT-QuIC testing of at least two biological samples with analyses by other available techniques.

### Surrogate Biomarkers

Although PrP^Sc^ is the most specific biochemical marker in TSEs, it is not the sole one. Surrogate biomarkers have been studied for both differential diagnosis and prognosis, especially in CJD cases. The search for biomarkers capable of predicting disease onset is especially important for patients with genetic forms, as well as for the inclusion of patients in clinical trials. Other proteins exploited for TSE diagnosis are 14-3-3 proteins, tau, neuron-specific enolase (NSE), astroglial protein S100B, α-synuclein, and neurofilament light chain protein (NFL) (Table 4). Among these, CSF 14-3-3 is a biomarker already considered in current guidelines (CDC’s Diagnostic Criteria for Creutzfeldt–Jakob Disease (CJD), 2018).

**TABLE 4:** Surrogate biomarkers of prion diseases.

Biomarker	Body fluid	Detection method	Sensitivity	Specificity	References
14-3-3	CSF	Western blotting, capture assay, ELISA	53%–100% (sCJD), 0%–97% (gCJD/FFI/GSS), 60%–75% (iCJD), 45%–58% (vCJD)	27%–100%	Hsich et al. (1996), Zerr et al. (1998, 2000a, b), Beaudry et al. (1999); Kenney et al. (2000), Lemstra et al. (2000); Aksamit et al. (2001), Green et al. (2001, 2002), Peoc’h et al. (2001); Otto et al. (2002), Geschwind et al. (2003); Van Everbroeck et al. (2003), Collins et al. (2006), Sanchez-Juan et al. (2006, 2007), Bahl et al. (2009), Gmitterová et al. (2009); Ladogana et al. (2009), Chohan et al. (2010); Stoeck et al. (2012), Sano et al. (2013); Schmitz et al. (2016), Hermann et al. (2018); Abu-Rumeileh et al. (2019), Llorens et al. (2020b)
tau	CSF	ELISA	75%–100% (sCJD), 0%–86% (gCJD/FFI/GSS), 53% (iCJD), 24%–80% (vCJD)	49%–100%	Otto et al. (1997, 2002), Green et al. (2001); Van Everbroeck et al. (2003), Sanchez-Juan et al. (2006, 2007), Bahl et al. (2009); Ladogana et al. (2009), Chohan et al. (2010); Hamlin et al. (2012), Stoeck et al. (2012); Sano et al. (2013), Steinacker et al. (2016), Abu-Rumeileh et al. (2018, 2019)
	Serum/Plasma	Simoa	57%–91% (sCJD/gCJD)	83%–97%	Steinacker et al. (2016), Thompson et al. (2018, 2020)
NSE	CSF	ELISA, TRACE	79%–85% (sCJD), 64% (gCJD), 52% (vCJD), 0% (GSS/FFI)	83%–92%	Zerr et al. (1995); Beaudry et al. (1999), Aksamit et al. (2001); Green et al. (2001), Bahl et al. (2009); Ladogana et al. (2009)
S100B	CSF	ELISA	65%–94% (sCJD), 87% (gCJD), 78% (vCJD), 50% (GSS), 20% (FFI)	76%–90%	Beaudry et al. (1999); Green et al. (2001), Ladogana et al. (2009); Chohan et al. (2010)
	Serum	ECL	78%–84% (sCJD/gCJD)	63%–81%	Otto et al. (1998); Steinacker et al. (2016)
α-Synuclein	CSF	ECL, ELISA	86%–98% (sCJD)	91%–98%	Llorens et al. (2017, 2018), Kruse et al. (2018); Schmitz et al. (2019)
NFL	CSF	ELISA	86%–97% (CJD/GSS)	43%–95%	Steinacker et al. (2016), Abu-Rumeileh et al. (2018, 2019)
	Serum/plasma	Simoa	93%–100% (sCJD/gCJD)	57%–100%	Steinacker et al. (2016), Thompson et al. (2018, 2020)

*CJD, Creutzfeldt–Jakob disease; ECL, electrochemiluminescence-based assays; ELISA, enzyme-linked immunosorbent assay; FFI, fatal familial insomnia; gCJD, genetic Creutzfeldt–Jakob disease; GSS, Gerstmann–Sträussler–Scheinker syndrome; iCJD, iatrogenic Creutzfeldt–Jakob disease; NFL, neurofilament light chain protein; NSE, neuron-specific enolase; sCJD, sporadic Creutzfeldt–Jakob disease; Simoa, single molecule array; TRACE, time-resolved amplified cryptate emission; vCJD, variant Creutzfeldt–Jakob disease.*

14-3-3 proteins are indicators of neuronal cell loss and can be detected in CSF samples by immunoblotting (Hsich et al., 1996), capture assay (Peoc’h et al., 2001), or enzyme-linked immunosorbent assay (ELISA) (Kenney et al., 2000). Measurement of CSF 14-3-3 has sensitivity values of 53%–100% for sCJD, 0%–97% for genetic prion diseases (gCJD, GSS, and FFI), 60%–70% for iCJD, and 45%–58% for vCJD. Numerous features account for these highly variable values: disease form (sCJD, gCJD, vCJD, iCJD, GSS, FFI), disease subtype (MM1, MM2, MV1, MV2, VV1, VV2), patient’s age, disease duration, and method used for quantification (Zerr et al., 2000b; Van Everbroeck et al., 2003; Collins et al., 2006; Gmitterová et al., 2009). Specificity values range from 27% to 100% since 14-3-3 elevation is not specific for TSEs. Acute neurological events (e.g., encephalitis, stroke, and epileptic seizures), brain tumor, and other neurodegenerative diseases also result in neuronal death, leading to an increase in 14-3-3 (Chohan et al., 2010; Stoeck et al., 2012). However, sensitivity and specificity have been shown to increase with disease duration in sCJD (Chohan et al., 2010).

An increase in CSF tau is typically an indicator of neuronal death associated with Alzheimer’s disease and can be measured by ELISA (Jensen et al., 1995). However, CSF tau levels are also increased in CJD and therefore have been extensively used for TSE diagnosis (Otto et al., 1997; Bahl et al., 2009; Stoeck et al., 2012; Abu-Rumeileh et al., 2019). Measurement of CSF tau has sensitivity values of 75%–100% for sCJD, 0%–86% for genetic prion diseases (gCJD, GSS, and FFI), 53% for iCJD, and 24%–80% for vCJD, while specificity values range from 49% to 100%. Similarly to 14-3-3, tau sensitivity is limited by disease form and subtype, patient’s age, and disease progression, while specificity is restricted by overlap with other diseases (Van Everbroeck et al., 2003; Hamlin et al., 2012; Stoeck et al., 2012; Cohen et al., 2016). CSF tau levels—along with information on patient’s age, sex, and codon 129 polymorphism—has been recently used to build the first prognostic model to predict the survival time of sCJD individuals with moderate to good accuracy (Llorens et al., 2020a).

The detection of elevated levels of the proteins NSE and S100B in CSF samples from CJD patients have also been reported (Zerr et al., 1995; Beaudry et al., 1999; Aksamit et al., 2001; Bahl et al., 2009; Ladogana et al., 2009). Measurement of CSF NSE and S100B has respective sensitivity values of 79%–85% and 65%–94% for sCJD, 0%–64% and 20%–87% for genetic prion diseases (gCJD, GSS, and FFI), and 52% and 78% for vCJD, while specificity values are 83–92% for NSE and 76%–90% for S100B. The combination of two or three biomarkers among 14-3-3, tau, NSE, and S100B significantly increased specificity, but at the cost of reduced sensitivity (Aksamit et al., 2001; Bahl et al., 2009; Chohan et al., 2010). However, in relation to 14-3-3 and tau, NSE and S100B alone showed no particular advantage and, therefore, have been much less explored for TSE diagnosis.

More recent studies reported that sCJD patients’ CSF also contains high levels of α-synuclein (Llorens et al., 2017, 2018; Kruse et al., 2018; Schmitz et al., 2019). Measurement of this protein had a sensitivity of 86%–98% and a specificity of 91%–98%, which are consistently high values in comparison to those of 14-3-3, tau, NSE, and S100B. These findings highlight α-synuclein as a very accurate surrogate biomarker in TSEs, but further studies are still necessary to reinforce the diagnostic utility of this protein.

Transmissible spongiform encephalopathy patients present elevated NFL in CSF as a result of neuroaxonal degeneration (Steinacker et al., 2016; Abu-Rumeileh et al., 2018, 2019). Measurement of CSF NFL has a sensitivity of 86%–97% for different TSE forms (sCJD, gCJD, and GSS) and specificity of 43%–95%. Increased tau and NFL levels can also be detected in TSE patients’ serum or plasma (Steinacker et al., 2016; Thompson et al., 2018, 2020). In sCJD and gCJD patients, measurement of blood tau and NFL has a respective sensitivity of 57%–91% and 93%–100% and specificity of 83–97% and 57%–100%. Blood tau and CSF tau display no significant difference in sensitivity and specificity, while blood NFL is slightly more accurate than CSF NFL.

Since serum and plasma samples are more easily obtained than CSF samples, these blood biomarkers also represent very useful tools for TSE diagnosis. Blood tau and NFL levels also increase in other neurological diseases and correlate with worsening of symptoms (Weydt et al., 2016; Mielke et al., 2017; Foiani et al., 2018), but they are generally higher in CJD patients (Thompson et al., 2020). Furthermore, blood tau concentration correlates with disease progression in sCJD patients and, thus, can be used to predict survival time (Thompson et al., 2018; Staffaroni et al., 2019). The analysis of plasma from patients carrying *PRNP* mutations associated with slowly progressive disease showed that NFL levels increase before the onset of symptoms (2 years before), suggesting that it may constitute a prodromal marker (Thompson et al., 2020). However, this interesting line of research will require additional studies with other populations.

Total PrP (t-PrP) has recently arisen as another potential biomarker. Since the concentration of t-PrP in CSF decreases as disease progresses (Villar-Piqué et al., 2019), it has been suggested as a pharmacodynamic biomarker (Minikel et al., 2019; Vallabh et al., 2019). CSF t-PrP concentration has been shown to be stable during 2 years of follow up, but none of the patients tested developed disease during the time frame of the research (Vallabh et al., 2020). The time point at which t-PrP levels start to decline and PrP^Sc^ levels start to increase in CSF is still unknown. Therefore, unveiling these events is of paramount importance for understanding disease progression. Additionally, elevated t-PrP levels have been detected in the plasma of sCJD and genetic TSE patients as a probable consequence of blood-brain barrier impairment (Llorens et al., 2020c). In this study, plasma t-PrP correlated with CSF biomarkers of neuronal death, but not with CSF t-PrP.

The diversity of TSE forms and subtypes poses a great challenge for defining a qualitative and quantitative standard for all the surrogate biomarkers reviewed here. As none of these approaches can undoubtedly distinguish human prion diseases from other neurological disorders, all these biomarkers should be analyzed with caution and always in combination with other methods, such as clinical evaluation, bioimaging (MRI) analysis, and EEG assessment (Hamlin et al., 2012).

### Electroencephalography (EEG)

Transmissible spongiform encephalopathy diagnoses can be supported by the appearance of periodic sharp wave complexes (PSWCs)—typical bilateral periodic discharges with a frequency of 0.5 to 2 per second—in EEG output. However, EEG findings alone are not specific for CJD, since PSWCs have been reported in patients with Alzheimer’s disease, vascular dementia, Lewy body disease, and voltage-gated potassium channel complex antibodies (VGKC-cAbs) encephalitis (Wieser et al., 2006; Savard et al., 2016). PSWCs are found in 60% to 70% of sCJD patients and 75% of fCJD patients, while they are rarely found in GSS and FFI cases and never in vCJD cases. The use of benzodiazepines for epilepsy has been shown to mask EEG findings. PSWCs appear months after the symptoms have started, and patients tend to recover a few weeks after their appearance. Meanwhile, bilateral frontal intermittent rhythmical delta activity (FIRDA) and a slowing of EEG background activity have been recorded in the early stages of CJD (Hansen et al., 1998; Manix et al., 2015). The absence of PSWC in EEG data does not exclude a diagnosis of sCJD, and typical EEG findings may not be observed in the terminal stage of the disease. Patients with CJD can also exhibit diffuse, non-specific slow EEG background patterns, which can also be observed in encephalopathy of various neurodegenerative diseases other than CJD. Hence, the diagnosis of sCJD should depend on a combination of EEG with MRI, CSF analysis, and clinical findings (Kwon and Kwon, 2019).

### Bioimaging

Neuroimaging tests are an accessible and non-invasive diagnostic tool for TSEs (Ortega-Cubero et al., 2013). Some neuroimaging techniques to help clinical neurologists provide a definite diagnosis have been extensively evaluated. In particular, CJD diagnosis has employed MRI, computed tomography (CT), radionuclide techniques using positron emission tomography (PET), and single-photon emission tomography (SPECT) with different radiopharmaceuticals (Caobelli et al., 2015). In clinical practice, the probable diagnosis of TSEs relies on clinical features combined with the results of at least one paraclinical test (EEG, CSF analysis, and/or MRI abnormalities).

Diffusion-weighted imaging (DWI), fluid-attenuated inversion recovery (FLAIR), and apparent diffusion coefficient (ADC) are MRI sequences used to image the brain. High-intensity patterns in FLAIR, hyperintensity signals in DWI (the most sensitive sequence), and restricted diffusion in the ADC map gray matter regions give a positive predictive value for TSEs, with sensitivity and specificity values of 80% for detecting CJD (Gibson et al., 2018). CJD diagnostic criteria by bioimaging includes the presence of abnormal signal in the caudate and putamen, and MRI signal hyperintensities in the cortex of at least two lobes, excluding the frontal lobes on DWI and FLAIR for sCJD (Zerr et al., 2009; Bizzi et al., 2020). A recent study included a new criterion (index test) for diffusion MRI for the diagnosis of sCJD, being at least one positive brain region in addition to the current criteria (Bizzi et al., 2020). Analysis of diffusion MRI including the index test from ∼1,400 patients with suspected sCJD was superior to that of current standard criteria and comparable to second generation RT-QuIC (Bizzi et al., 2020).

Between 57% and 80% of sCJD patients show hyperintensity in the basal ganglia or cortex, with some also showing hyperintensity in the thalamus. MRI results are usually normal in patients with GSS and FFI, though some FFI patients show increased quantitative ADC and myoinositol in the thalamus. Quantitative MRI changes were also reported for FFI, characterized by increased mean diffusivity in the thalamus and cerebellum resultant from volumetric and diffusion tensor imaging (DTI) changes in the patient’s brain (Grau-Rivera et al., 2017). In 90% of cases, vCJD shows a pulvinar sign in which the posterior third of the thalamus has a higher signal than other basal ganglia (Manix et al., 2015; Fragoso et al., 2017). MRI signal changes are correlated with disease duration and with the degree of spongiosis (Eisenmenger et al., 2016). The strict application of diagnostic criteria and careful interpretation of MRI output remain the most recommended approach to *in vivo* diagnosis of sCJD (Fragoso et al., 2017). In the context of clinical suspicion of TSEs, however, CT does remain useful as a first-line test to exclude other diagnoses that may have similar clinical features (Letourneau-Guillon et al., 2012).

Positron emission tomography (PET) is not a paraclinical test included in the guidelines for TSE diagnosis, but it has recently been tested with a small group of patients. CJD patients showed hypometabolism in the frontal and parietal cortex using [18F] fluoro-2-deoxy-D-glucose (FDG-PET), suggesting a correlation between clinical results and PET findings (Renard et al., 2017). In a different group of patients, hypermetabolism was observed in the limbic and mesolimbic systems (Mente et al., 2017). Hypometabolism in brain regions is consistent with neuropathology, but it is not an exclusive characteristic of prion diseases (Higuchi et al., 2000).

Amyloid tracers may also be used with PET to diagnose neurodegenerative diseases, but the selectivity of these tracers remains a topic of discussion and requires improvement. Therefore, this technique is still limited for the differential diagnosis of these diseases, though it has yet to be explored in detail in living patients.

### Immunoassays

Immunoassays exploit the structural differences between PrP^C^ and PrP^Sc^ and rely on the specific interaction of monoclonal antibodies (mAbs) with PrP^Sc^ (Lukan et al., 2013). Although with potential application in diagnosis, immunoassays developed for analyzing body fluids, such as blood, CSF, or urine, have certain limitations. Unlike diagnostic studies using *in vitro* cell-free PrP conversion assays, reports of immunoassays have been restricted to blood samples not endogenously infected with PrP^Sc^ or from symptomatic vCJD patients. Almost all blood-based immunoassays developed to date rely on the detection of PrP^Sc^. The major issue in blood detection systems is an extremely small amount of PrP^Sc^ combined with a high background PrP^C^ concentration (Abdel-Haq, 2015). For this reason, a PrP^Sc^ enrichment step is usually required in these assays (Lukan et al., 2013). The solid-state binding matrix assay relies on the selective adsorption of PrP^Sc^, thus, no PK pre-treatment of samples is required in this assay (Properzi and Pocchiari, 2013), being also adapted for urine assessment (Luk et al., 2016). This approach has led to the establishment of a prototype blood test for the diagnosis of vCJD with high sensitivity and specificity (Edgeworth et al., 2011), and was shown to be more efficient than immunoprecipitation with antibodies for detecting PrP^Sc^ in human whole blood spiked with high dilutions of vCJD-infected brain homogenate (Abdel-Haq, 2015). Although initially promising, this test failed to identify patients with sCJD or other TSEs (Edgeworth et al., 2011) and asymptomatic vCJD individuals (Jackson et al., 2014; Abdel-Haq, 2015). In summary, all these approaches present severe limitations regarding efficient detection of PrP^Sc^ in a variety of body fluids and tissues, being not recommended for routine diagnosis of human TSEs.

## Guidelines for Diagnosis

The WHO prepared a report in 1998, updated in 2003, defining the diagnostic criteria for CJD. However, much research has been carried out in the intervening years, and new diagnostic tools have been developed and tested with great success. The Centers for Disease Control and Prevention (CDC), an agency of the United States Department of Health and Human Services, updated the clinical diagnostic criteria for sCJD to include MRI brain scans and RT-QuIC analysis for the probable diagnosis of sCJD (Table 5).

**TABLE 5:** Current guidelines for sCJD diagnosis.

Diagnostic	Signals and symptoms	
	^∗^WHO, 2003	^∗∗^CDC, 2018
Possible	Progressive dementia; **AND** at least two of the following four clinical features: myoclonus, cerebellar or visual disturbance, pyramidal/extrapyramidal dysfunction, akinetic mutism; **AND** unknown or atypical EEG; **AND** disease duration less than two years.	Progressive dementia; **AND** at least two of the following four clinical features: myoclonus, cerebellar or visual disturbance, pyramidal/extrapyramidal dysfunction, akinetic mutism; **AND** no positive result for any of the four tests that would classify a case as “probable”; **AND** disease duration less than two years; **AND** no alternative diagnosis from routine investigations.
Probable	Progressive dementia; **AND** at least two of the following four clinical features: myoclonus, cerebellar or visual disorder, pyramidal/extrapyramidal dysfunction, akinetic mutism; **AND** typical EEG regardless of disease duration; **AND/OR** positive CSF 14-3-3 assay **and** disease duration to death less than two years; **AND** no alternative diagnosis from routine investigations.	Neuropsychiatric disorder **and** positive RT-QuIC in CSF or other samples; **OR** rapidly progressive dementia **and** at least two of the following four clinical features: myoclonus, cerebellar or visual disorder, pyramidal/extrapyramidal dysfunction, akinetic mutism; **AND** a positive result on at least one of the following tests: typical EEG regardless of disease duration, positive CSF 14-3-3 assay with disease duration less than two years, high signal in caudate/putamen on MRI brain scan or at least two cortical regions (temporal, parietal, occipital) either on DWI or FLAIR; **AND** no alternative diagnosis from routine investigations.
Definite	Neuropathological confirmation; **AND/OR** detection of ^*res*^PrP by immunochemistry **or** immunoblotting; **AND/OR** observation of PrP^*Sc*^ fibrils.	Neuropathological confirmation; **AND/OR** detection of ^*res*^PrP by immunochemistry **or** immunoblotting; **AND/OR** observation of PrP^*Sc*^ fibrils.

*^∗^Reference: Brown et al. (2003). ^∗∗^Reference: CDC’s Diagnostic Criteria for Creutzfeldt-Jakob Disease (CJD), 2018.*

The diagnosis of iCJD was defined as a progressive cerebellar syndrome in a patient treated with pituitary hormone receptor derived from human cadavers; or in a patient diagnosed with sporadic CJD with a recognized exposure risk—e.g., previous neurosurgery with dura mater graft. Genetic CJD is classified as definite or probable CJD plus definite or probable CJD in a first-degree relative; and/or neuropsychiatric disorder plus disease-specific PrP gene mutation (Brown et al., 2003; CDC’s Diagnostic Criteria for Creutzfeldt–Jakob Disease (CJD), 2018).

One characteristic that distinguishes vCJD from sCJD is its prominent tropism for lymphoid organs, such as the tonsils. The analysis of PrP extracted from amygdala biopsy tissue appears to provide a sensitive and specific method for the diagnosis of vCJD in the appropriate clinical context (Araújo, 2013). However, due to the invasive nature of this test, it should only be performed in patients who meet the clinical criteria for vCJD, but where MRI data lacks the characteristic pulmonary sign. The detection of 14-3-3 protein in the CSF is not a sensitive marker for vCJD (Araújo, 2013). Definitive cases are diagnosed by visualization of amyloid plaques surrounded by vacuoles in both the cerebellum and cerebrum, called florid plaques, and by PrP deposition shown by immunohistochemistry (CDC’s Diagnostic Criteria for Creutzfeldt–Jakob Disease (CJD), 2018). Suspected cases are identified based on the age of onset of the disease (<50 years), psychiatric symptoms (such as frank pain and/or dysesthesia), neurologic signs (at least two of the following: poor coordination, myoclonus, chorea, hyperreflexia, or visual signs), duration of illness of over 6 months, and no history for iCJD or gCJD.

Because of its genetic character, the diagnosis of GSS is limited to the demonstration of mutations in the *PRNP* gene. This seems to be a sensitive and highly specific way to diagnose GSS, but neuropathology, although less used in practice, can also be useful in a postmortem scenario (Araújo, 2013).

Finally, the diagnosis of FFI is first suggested by rapidly progressive cognitive impairment (dementia), along with changes in behavior or mood, ataxia, and sleep disorders. Additional diagnosis may include a sleep study. Given its etiology, genetic testing confirms the diagnosis (Wu et al., 2018).

## Challenges

Human prion diseases are rare, with an annual incidence of around 1–2 cases per million individuals worldwide, and around five cases per million after age 65 (Holman et al., 2010). These diseases are very intriguing and can have a quite distinct clinical presentation and causes (sporadic, inherited, and acquired). This heterogeneity poses challenges for diagnoses. Interestingly, all prion diseases involve the conversion and aggregation of PrP^C^ into its pathological form, PrP^Sc^. Therefore, it is no wonder that the detection of PrP^Sc^ is used as the gold standard for a definite diagnosis.

The detection of PrP^Sc^ in patient samples is traditionally performed by neuropathological inspection of brain tissue, which is a criterion for a definite diagnosis (Brown et al., 2003). However, this assessment is done most often postmortem due to the invasiveness of tissue collection. Clinical and paraclinical antemortem evaluations are not considered sufficient to define the final diagnosis. However, with continued technological advances, there has been a marked improvement in several tests in recent years, contributing to the interpretation of cases.

The last decade has seen the development of the RT-QuIC assay, which can now detect the presence of PrP^Sc^. This technique has several advantages, including:

(1) It can be used on more accessible samples, such as CSF;
(2) It exhibits high sensitivity and specificity, making other biochemical tests obsolete;
(3) It can detect PrP^Sc^ in samples from different types of TSEs;
(4) It involves a relatively simple method, employing materials and equipment that are more accessible and easier to maintain;
(5) A growing number of centers around the world have independently tested, validated, and used this technique to investigate large cohorts of patients;
(6) It is being adapted for the diagnosis of other neurodegenerative diseases, such as Parkinson’s disease, tauopathies, and others.

Given these advantages, the CDC has included RT-QuIC as a parameter to characterize TSE diagnosis as probable (CDC’s Diagnostic Criteria for Creutzfeldt–Jakob Disease (CJD), 2018). WHO guidelines do not yet include this parameter and should be updated to encourage its implementation in more facilities that can carry out these tests. The combination of (*i*) clinical evaluation, (*ii*) RT-QuIC analyses of two biological samples, and (*iii*) testing with at least one other technique—such as MRI, EEG, or 14-3-3 detection—should be considered internationally for a definite antemortem diagnosis, given that such combinations have provided diagnoses with 100% or nearly 100% sensitivity and specificity (Bongianni et al., 2017; Hermann et al., 2018; Rudge et al., 2018; Fiorini et al., 2020).

Furthermore, the RT-QuIC assay has been adapted in the last few years for detecting α-synuclein aggregates/fibrils and tau filaments in biological samples (Ferreira and Caughey, 2020). Therefore, the growth in RT-QuIC-based TSE diagnosis would certainly pave the way for RT-QuIC utilization in the early diagnosis of other neurodegenerative disorders, such as synucleinopathies (e.g., Parkinson’s disease and dementia with Lewy bodies) and tauopathies (e.g., Alzheimer’s disease and chronic traumatic encephalopathy.

Even with all the advances achieved to date in TSE diagnosis, most patients present the first typical clinical signs of the disease very late, when the degeneration is already quite advanced and irreversible. Regular clinical evaluation is only initiated at this point, resulting in therapeutic options that are, at best, symptomatic. Therefore, a significant challenge is to further increase the sensitivity and accuracy of testing to allow early antemortem diagnosis and to relate the findings to the progression of the disease. Another challenge is to obtain a diagnosis at pre-symptomatic stages of genetic forms of TSEs, which would require the combination of genotyping and the examination of samples using the assays discussed in this review. If an early diagnosis can be achieved, this opens the door to novel therapeutic strategies being tested and used successfully in the future.

## Author Contributions

YC, TCRGV, and LMA contributed to the conception and design of the study. SCR wrote the first draft of the manuscript. TCRGV, YC, LMA, and PBG wrote sections of the manuscript. LMA produced all figures. All authors contributed to the manuscript revision and read and approved the submitted version.

## Conflict of Interest

The authors declare that the research was conducted in the absence of any commercial or financial relationships that could be construed as a potential conflict of interest.

## References

[B1] Abdel-Haq H. (2015). Factors intrinsic and extrinsic to blood hamper the development of a routine blood test for human prion diseases. *J. Gen. Virol.* 96 479–493. 10.1099/vir.0.070979-0 25389187

[B2] Abu-Rumeileh S. Baiardi S. Polischi B. Mammana A. Franceschini A. Green A. (2019). Diagnostic value of surrogate CSF biomarkers for Creutzfeldt–Jakob disease in the era of RT-QuIC. *J. Neurol.* 266 3136–3143. 10.1007/s00415-019-09537-0 31541342

[B3] Abu-Rumeileh S. Capellari S. Stanzani-Maserati M. Polischi B. Martinelli P. Caroppo P. (2018). The CSF neurofilament light signature in rapidly progressive neurodegenerative dementias. *Alzheimer’s Res. Ther.* 10:3. 10.1186/s13195-017-0331-1 29368621 PMC5784714

[B4] Acquatella-Tran Van Ba I. Imberdis T. Perrier V. (2013). From prion diseases to prion-like propagation mechanisms of neurodegenerative diseases. *Int. J. Cell Biol.* 2013:975832. 10.1155/2013/975832 24222767 PMC3810426

[B5] Agrimi U. Nonno R. Dell’Omo G. Di Bari M. A. Conte M. Chiappini B. (2008). Prion protein amino acid determinants of differential susceptibility and molecular feature of prion strains in mice and voles. *PLoS Pathog.* 4:e1000113. 10.1371/journal.ppat.1000113 18654630 PMC2453331

[B6] Aguzzi A. Glatzel M. Montrasio F. Prinz M. Heppner F. L. (2001). Interventional strategies against prion diseases. *Nat. Rev. Neurosci.* 2 745–749. 10.1038/35094590 11584312

[B7] Aguzzi A. Heikenwalder M. Polymenidou M. (2007). Insights into prion strains and neurotoxicity. *Nat. Rev. Mol. Cell Biol.* 8 552–561. 10.1038/nrm2204 17585315

[B8] Aksamit A. J. Preissner C. M. Homburger H. A. (2001). Quantitation of 14-3-3 and neuron-specific enolase proteins in CSF in Creutzfeldt-Jakob disease. *Neurology* 57 728–730. 10.1212/WNL.57.4.728 11524493

[B9] Andréoletti O. Litaise C. Simmons H. Corbière F. Lugan S. Costes P. (2012). Highly efficient prion transmission by blood transfusion. *PLoS Pathog.* 8:e1002782. 10.1371/journal.ppat.1002782 22737075 PMC3380953

[B10] Annus Á Csáti A. Vécsei L. (2016). Prion diseases: new considerations. *Clin. Neurol. Neurosurg.* 150 125–132. 10.1016/j.clineuro.2016.09.006 27656779

[B11] Araújo A. Q. C. (2013). Doenças priônicas. *Arq. Neuropsiquiatr.* 71 731–737. 10.1590/0004-282X201301461 24141515

[B12] Atarashi R. Moore R. A. Sim V. L. Hughson A. G. Dorward D. W. Onwubiko H. A. (2007). Ultrasensitive detection of scrapie prion protein using seeded conversion of recombinant prion protein. *Nat. Methods* 4 645–650. 10.1038/nmeth1066 17643109

[B13] Atarashi R. Satoh K. Sano K. Fuse T. Yamaguchi N. Ishibashi D. (2011). Ultrasensitive human prion detection in cerebrospinal fluid by real-time quaking-induced conversion. *Nat. Med.* 17 175–178. 10.1038/nm.2294 21278748

[B14] Atarashi R. Wilham J. M. Christensen L. Hughson A. G. Moore R. A. Johnson L. M. (2008). Simplified ultrasensitive prion detection by recombinant PrP conversion with shaking. *Nat. Methods* 5 211–212. 10.1038/nmeth0308-211 18309304

[B15] Ayers J. I. Prusiner S. B. (2020). Prion protein — mediator of toxicity in multiple proteinopathies. *Nat. Rev. Neurol.* 16 187–188. 10.1038/s41582-020-0332-8 32123368

[B16] Bahl J. M. C. Heegaard N. H. H. Falkenhorst G. Laursen H. Høgenhaven H. Mølbak K. (2009). The diagnostic efficiency of biomarkers in sporadic Creutzfeldt-Jakob disease compared to Alzheimer’s disease. *Neurobiol. Aging* 30 1834–1841. 10.1016/j.neurobiolaging.2008.01.013 18339451

[B17] Baral P. K. Yin J. Aguzzi A. James M. N. G. (2019). Transition of the prion protein from a structured cellular form (PrPC) to the infectious scrapie agent (PrPSc). *Protein Sci.* 28 2055–2063. 10.1002/pro.3735 31583788 PMC6863700

[B18] Beaudry P. Cohen P. Brandel J. P. Delasnerie-Lauprêtre N. Richard S. Launay J. M. (1999). 14-3-3 Protein, neuron-specific enolase, and S-100 protein in cerebrospinal fluid of patients with Creutzfeldt-Jakob disease. *Dement. Geriatr. Cogn. Disord.* 10 40–46. 10.1159/000017095 10068257

[B19] Bellon A. Seyfert-Brandt W. Lang W. Baron H. Gröner A. Vey M. (2003). Improved conformation-dependent immunoassay: suitability for human prion detection with enhanced sensitivity. *J. Gen. Virol.* 84 1921–1925. 10.1099/vir.0.18996-0 12810888

[B20] Bendheim P. E. Barry R. A. Dearmond S. J. Stites D. P. Prusiner S. B. (1984). Antibodies to a scrapie prion protein. *Nature* 310 418–421. 10.1038/310418a0 6431296

[B21] Bessen R. A. Kocisko D. A. Raymond G. J. Nandan S. Lansbury P. T. Caughey B. (1995). Non-genetic propagation of strain-specific properties of scrapie prion protein. *Nature* 375 698–700. 10.1038/375698a0 7791905

[B22] Bizzi A. Pascuzzo R. Blevins J. Grisoli M. Lodi R. Moscatelli M. E. M. (2020). Evaluation of a new criterion for detecting prion disease with diffusion magnetic resonance Imaging. *JAMA Neurol.* 77 1–9. 10.1001/jamaneurol.2020.1319 32478816 PMC7265127

[B23] Bongianni M. Orrù C. Groveman B. R. Sacchetto L. Fiorini M. Tonoli G. (2017). Diagnosis of human prion disease using real-time quaking-induced conversion testing of olfactory mucosa and cerebrospinal fluid samples. *JAMA Neurol.* 74 155–162. 10.1001/jamaneurol.2016.4614 27942718

[B24] Bougard D. Brandel J. P. Bélondrade M. Béringue V. Segarra C. Fleury H. (2016). Detection of prions in the plasma of presymptomatic and symptomatic patients with variant Creutzfeldt-Jakob disease. *Sci. Transl. Med.* 8:370ra182. 10.1126/scitranslmed.aag1257 28003547

[B25] Brown P. Brunk C. Budka H. Cervenakova L. Collie D. Green A. (2003). *). WHO Manual for Surveillance of Human Transmissible Spongiform Encephalopathies, Including Variant Creutzfeldt-Jakob Disease.* Geneva: World Health Organization.

[B26] Caobelli F. Cobelli M. Pizzocaro C. Pavia M. Magnaldi S. Guerra U. P. (2015). The role of neuroimaging in evaluating patients affected by creutzfeldt-jakob disease: a systematic review of the literature. *J. Neuroimaging* 25 2–13. 10.1111/jon.12098 24593302

[B27] Castilla J. Saá P. Soto C. (2004). “Cyclic Amplification of Prion Protein Misfolding,” in *Techniques in Prion Research*, eds Lehmann S. Grassi J. (Basel: Birkhäuser), 198–213. 10.1007/978-3-0348-7949-1_14

[B28] Castilla J. Saá P. Soto C. (2005). Detection of prions in blood. *Nat. Med.* 11 982–985. 10.1038/nm1286 16127436

[B29] Caughey B. (2000). Formation of protease-resistant prion protein in cell-free systems. *Curr. Issues Mol. Biol.* 2 95–101. 11471561

[B30] Caughey B. Baron G. S. Chesebro B. Jeffrey M. (2009). Getting a grip on prions: oligomers, amyloids, and pathological membrane interactions. *Annu. Rev. Biochem.* 78 177–204. 10.1146/annurev.biochem.78.082907.145410 19231987 PMC2794486

[B31] CDC’s Diagnostic Criteria for Creutzfeldt–Jakob Disease (CJD) (2018). *Centers Dis. Control Prev.* Available online at: https://www.cdc.gov/prions/cjd/diagnostic-criteria.html (accessed September 22, 2020).

[B32] Chen C. Dong X. P. (2016). Epidemiological characteristics of human prion diseases. *Infect. Dis. Poverty* 5:47. 10.1186/s40249-016-0143-8 27251305 PMC4890484

[B33] Chesebro B. (2003). Introduction to the transmissible spongiform encephalopathies or prion diseases. *Br. Med. Bull.* 66 1–20. 10.1093/bmb/dg66.001 14522845

[B34] Chiti F. Dobson C. M. (2006). Protein misfolding, functional amyloid, and human disease. *Annu. Rev. Biochem.* 75 333–366. 10.1146/annurev.biochem.75.101304.123901 16756495

[B35] Chitravas N. Jung R. S. Kofskey D. M. Blevins J. E. Gambetti P. Leigh R. J. (2011). Treatable neurological disorders misdiagnosed as Creutzfeldt-Jakob disease. *Ann. Neurol.* 70 437–444. 10.1002/ana.22454 21674591 PMC3170496

[B36] Chohan G. Pennington C. Mackenzie J. M. Andrews M. Everington D. Will R. G. (2010). The role of cerebrospinal fluid 14-3-3 and other proteins in the diagnosis of sporadic Creutzfeldt-Jakob disease in the UK: a 10-year review. *J. Neurol. Neurosurg. Psychiatry* 81 1243–1248. 10.1136/jnnp.2009.197962 20855493

[B37] Cobb N. J. Surewicz W. K. (2009). Prion diseases and their biochemical mechanisms. *Biochemistry* 48 2574–2585. 10.1021/bi900108v 19239250 PMC2805067

[B38] Cohen O. S. Chapman J. Korczyn A. D. Warman-Alaluf N. Nitsan Z. Appel S. (2016). CSF tau correlates with CJD disease severity and cognitive decline. *Acta Neurol. Scand.* 133 119–123. 10.1111/ane.12441 26014384

[B39] Colby D. W. Prusiner S. B. (2011). De novo generation of prion strains. *Nat. Rev. Microbiol.* 9 771–777. 10.1038/nrmicro2650 21947062 PMC3924856

[B40] Colby D. W. Zhang Q. Wang S. Groth D. Legname G. Riesner D. (2007). Prion detection by an amyloid seeding assay. *Proc. Natl. Acad. Sci. U.S.A.* 104 20914–20919. 10.1073/pnas.0710152105 18096717 PMC2409241

[B41] Collins S. J. Sanchez-Juan P. Masters C. L. Klug G. M. Van Duijn C. Poleggi A. (2006). Determinants of diagnostic investigation sensitivities across the clinical spectrum of sporadic Creutzfeldt-Jakob disease. *Brain* 129 2278–2287. 10.1093/brain/awl159 16816392

[B42] Concha-Marambio L. Pritzkow S. Moda F. Tagliavini F. Ironside J. W. Schulz P. E. (2016). Detection of prions in blood from patients with variant Creutzfeldt-Jakob disease. *Sci. Transl. Med.* 8:370ra183. 10.1126/scitranslmed.aaf6188 28003548 PMC5538786

[B43] Corbett G. T. Wang Z. Hong W. Colom-Cadena M. Rose J. Liao M. (2020). PrP is a central player in toxicity mediated by soluble aggregates of neurodegeneration-causing proteins. *Acta Neuropathol.* 139 503–526. 10.1007/s00401-019-02114-9 31853635 PMC7035229

[B44] Cracco L. Appleby B. S. Gambetti P. (2018). Fatal familial insomnia and sporadic fatal insomnia. *Handb. Clin. Neurol.* 153 271–299. 10.1016/B978-0-444-63945-5.00015-5 29887141

[B45] Cramm M. Schmitz M. Karch A. Mitrova E. Kuhn F. Schroeder B. (2016). Stability and reproducibility underscore utility of RT-QuIC for diagnosis of Creutzfeldt-Jakob Disease. *Mol. Neurobiol.* 53 1896–1904. 10.1007/s12035-015-9133-2 25823511 PMC4789202

[B46] Cramm M. Schmitz M. Karch A. Zafar S. Varges D. Mitrova E. (2015). Characteristic CSF prion seeding efficiency in humans with prion diseases. *Mol. Neurobiol.* 51 396–405. 10.1007/s12035-014-8709-6 24809690 PMC4309904

[B47] Di Bari M. A. Chianini F. Vaccari G. Esposito E. Conte M. Eaton S. L. (2008). The bank vole (Myodes glareolus) as a sensitive bioassay for sheep scrapie. *J. Gen. Virol.* 89 2975–2985. 10.1099/vir.0.2008/005520-0 19008382

[B48] Di Bari M. A. Nonno R. Castilla J. D’Agostino C. Pirisinu L. Riccardi G. (2013). Chronic wasting disease in bank voles: characterisation of the shortest incubation time model for prion diseases. *PLoS Pathog.* 9:e1003219. 10.1371/journal.ppat.1003219 23505374 PMC3591354

[B49] Diack A. B. Ritchie D. L. Peden A. H. Brown D. Boyle A. Morabito L. (2014). Variably protease-sensitive prionopathy, a unique prion variant with inefficient transmission properties. *Emerg. Infect. Dis.* 20 1969–1979. 10.3201/eid2012.140214 25418327 PMC4257789

[B50] Edgeworth J. A. Farmer M. Sicilia A. Tavares P. Beck J. Campbell T. (2011). Detection of prion infection in variant Creutzfeldt-Jakob disease: a blood-based assay. *Lancet* 377 487–493. 10.1016/S0140-6736(10)62308-2 21295339

[B51] Eisenmenger L. Porter M. C. Carswell C. J. Thompson A. Mead S. Rudge P. (2016). Evolution of diffusion-weighted magnetic resonance imaging signal abnormality in sporadic Creutzfeldt-jakob disease, with histopathological correlation. *JAMA Neurol.* 73 76–84. 10.1001/jamaneurol.2015.3159 26569479 PMC5837002

[B52] Feinstein A. R. (1975). XXXI. On the sensitivity, specificity, and discrimination of diagnostic tests. *Clin. Pharmacol. Ther.* 17 104–116. 10.1002/cpt1975171104 1122664

[B53] Ferreira N. D. C. Caughey B. (2020). Proteopathic seed amplification assays for neurodegenerative disorders. *Clin. Lab. Med.* 40 257–270. 10.1016/j.cll.2020.04.002 32718498 PMC9392962

[B54] Fiorini M. Iselle G. Perra D. Bongianni M. Capaldi S. Sacchetto L. (2020). High diagnostic accuracy of RT-QuIC assay in a prospective study of patients with suspected sCJD. *Int. J. Mol. Sci.* 21:880. 10.3390/ijms21030880 32019068 PMC7038328

[B55] Foiani M. S. Woollacott I. O. C. Heller C. Bocchetta M. Heslegrave A. Dick K. M. (2018). Plasma tau is increased in frontotemporal dementia. *J. Neurol. Neurosurg. Psychiatry* 89 804–807. 10.1136/jnnp-2017-317260 29440230 PMC6204947

[B56] Forloni G. Roiter I. Tagliavini F. (2019). Clinical trials of prion disease therapeutics. *Curr. Opin. Pharmacol.* 44 53–60. 10.1016/j.coph.2019.04.019 31108459

[B57] Foutz A. Appleby B. S. Hamlin C. Liu X. Yang S. Cohen Y. (2017). Diagnostic and prognostic value of human prion detection in cerebrospinal fluid. *Ann. Neurol.* 81 79–92. 10.1002/ana.24833 27893164 PMC5266667

[B58] Fragoso D. C. Gonçalves Filho A. L. da M. Pacheco F. T. Barros B. R. Littig I. A. (2017). Imaging of Creutzfeldt-Jakob disease: imaging patterns and their differential diagnosis. *Radiographics* 37 234–257. 10.1148/rg.2017160075 28076012

[B59] Franceschini A. Baiardi S. Hughson A. G. McKenzie N. Moda F. Rossi M. (2017). High diagnostic value of second generation CSF RT-QuIC across the wide spectrum of CJD prions. *Sci. Rep.* 7:10655. 10.1038/s41598-017-10922-w 28878311 PMC5587608

[B60] Gambetti P. Kong Q. Zou W. Parchi P. Chen S. G. (2003). Sporadic and familial CJD: classification and characterisation. *Br. Med. Bull.* 66 213–239. 10.1093/bmb/66.1.213 14522861

[B61] Geschwind M. D. (2015). Prion Diseases. *Contin. Lifelong Learn. Neurol.* 21 1612–1638. 10.1212/CON.0000000000000251 26633779 PMC4879966

[B62] Geschwind M. D. Martindale J. Miller D. DeArmond S. J. Uyehara-Lock J. Gaskin D. (2003). Challenging the clinical utility of the 14-3-3 protein for the diagnosis of sporadic Creutzfeldt-Jakob disease. *Arch. Neurol.* 60 813–816. 10.1001/archneur.60.6.813 12810484

[B63] Gibson L. M. Chappell F. M. Summers D. Collie D. A. Sellar R. Best J. (2018). Post-mortem magnetic resonance imaging in patients with suspected prion disease: pathological confirmation, sensitivity, specificity and observer reliability. A national registry. *PLoS One* 13:e0201434. 10.1371/journal.pone.0201434 30086144 PMC6080765

[B64] Gmitterová K. Heinemann U. Bodemer M. Krasnianski A. Meissner B. Kretzschmar H. A. (2009). 14-3-3 CSF levels in sporadic Creutzfeldt-Jakob disease differ across molecular subtypes. *Neurobiol. Aging* 30 1842–1850. 10.1016/j.neurobiolaging.2008.01.007 18328602

[B65] Grau-Rivera O. Calvo A. Bargalló N. Monté G. C. Nos C. Lladó A. (2017). Quantitative magnetic resonance abnormalities in creutzfeldt-jakob disease and fatal insomnia. *J. Alzheimer’s Dis.* 55 431–443. 10.3233/JAD-160750 27662320

[B66] Green A. J. E. Ramljak S. Müller W. E. G. Knight R. S. G. Schröder H. C. (2002). 14-3-3 in the cerebrospinal fluid of patients with variant and sporadic Creutzfeldt-Jakob disease measured using capture assay able to detect low levels of 14-3-3 protein. *Neurosci. Lett.* 324 57–60. 10.1016/S0304-3940(02)00172-6 11983294

[B67] Green A. J. E. Thompson E. J. Stewart G. E. Zeidler M. McKenzie J. M. MacLeod M. A. (2001). Use of 14-3-3 and other brain-specific proteins in CSF in the diagnosis of variant Creutzfeldt-Jakob disease. *J. Neurol. Neurosurg. Psychiatry* 70 744–748. 10.1136/jnnp.70.6.744 11385008 PMC1737395

[B68] Groveman B. R. Kraus A. Raymond L. D. Dolan M. A. Anson K. J. Dorward D. W. (2015). Charge neutralization of the central lysine cluster in prion protein (PrP) promotes PrPSc-Like folding of recombinant PrP amyloids. *J. Biol. Chem.* 290 1119–1128. 10.1074/jbc.M114.619627 25416779 PMC4294479

[B69] Groveman B. R. Orrú C. D. Hughson A. G. Bongianni M. Fiorini M. Imperiale D. (2016). Extended and direct evaluation of RT-QuIC assays for Creutzfeldt-Jakob disease diagnosis. *Ann. Clin. Transl. Neurol.* 4 139–144. 10.1002/acn3.378 28168213 PMC5288466

[B70] Groveman B. R. Raymond G. J. Campbell K. J. Race B. Raymond L. D. Hughson A. G. (2017). Role of the central lysine cluster and scrapie templating in the transmissibility of synthetic prion protein aggregates. *PLoS Pathog.* 13:e1006623. 10.1371/journal.ppat.1006623 28910420 PMC5614645

[B71] Haley N. J. Richt J. A. (2017). Evolution of diagnostic tests for chronic wasting disease, a naturally occurring prion disease of cervids. *Pathogens* 6:35. 10.3390/pathogens6030035 28783058 PMC5617992

[B72] Hamlin C. Puoti G. Berri S. Sting E. Harris C. Cohen M. (2012). A comparison of tau and 14-3-3 protein in the diagnosis of Creutzfeldt-Jakob disease. *Neurology* 79 547–552. 10.1212/WNL.0b013e318263565f 22843257 PMC4098812

[B73] Hansen H. C. Zschocke S. Stürenburg H. J. Kunze K. (1998). Clinical changes and EEG patterns preceding the onset of periodic sharp wave complexes in Creutzfeldt-Jakob disease. *Acta Neurol. Scand.* 97 99–106. 10.1111/j.1600-0404.1998.tb00617.x 9517859

[B74] Hermann P. Laux M. Glatzel M. Matschke J. Knipper T. Goebel S. (2018). Validation and utilization of amended diagnostic criteria in Creutzfeldt-Jakob disease surveillance. *Neurology* 91 e331–e338. 10.1212/WNL.0000000000005860 29934424

[B75] Higuchi M. Tashiro M. Arai H. Okamura N. Hara S. Higuchi S. (2000). Glucose hypometabolism and neuropathological correlates in brains of dementia with Lewy bodies. *Exp. Neurol.* 162 247–256. 10.1006/exnr.2000.7342 10739631

[B76] Hill A. F. Desbruslais M. Joiner S. Sidle K. C. L. Gowland I. Collinge J. (1997). The same prion strain causes vCJD and BSE. *Nature* 389 448–450. 10.1038/38925 9333232

[B77] Holman R. C. Belay E. D. Christensen K. Y. Maddox R. A. Minino A. M. Folkema A. M. (2010). Human prion diseases in the United States. *PLoS One* 5:e8521. 10.1371/journal.pone.0008521 20049325 PMC2797136

[B78] Hsich G. Kenney K. Gibbs C. J. Lee K. H. Harrington M. G. (1996). The 14-3-3 brain protein in cerebrospinal fluid as a marker for transmissible spongiform encephalopathies. *N. Engl. J. Med.* 335 924–930. 10.1056/NEJM199609263351303 8782499

[B79] Hunter N. Foster J. Chong A. McCutcheon S. Parnham D. Eaton S. (2002). Transmission of prion diseases by blood transfusion. *J. Gen. Virol.* 83 2897–2905. 10.1099/0022-1317-83-11-2897 12388826

[B80] Igel-Egalon A. Béringue V. Rezaei H. Sibille P. (2018). Prion strains and transmission barrier phenomena. *Pathogens* 7:5. 10.3390/pathogens7010005 29301257 PMC5874731

[B81] Ironside J. W. Ritchie D. L. Head M. W. (2017). Prion diseases. *Handb. Clin. Neurol.* 145 393–403. 10.1016/B978-0-12-802395-2.00028-6 28987186

[B82] Iwasaki Y. (2017). Creutzfeldt-Jakob disease. *Neuropathology* 37 174–188. 10.1111/neup.12355 28028861

[B83] Jackson G. S. Burk-Rafel J. Edgeworth J. A. Sicilia A. Abdilahi S. Korteweg J. (2014). Population screening for variant Creutzfeldt-Jakob disease using a novel blood test: diagnostic accuracy and feasibility study. *JAMA Neurol.* 71 421–428. 10.1001/jamaneurol.2013.6001 24590363 PMC4158718

[B84] Jensen M. Basun H. Lannfelt L. (1995). Increased cerebrospinal fluid tau in patients with Alzheimer’s disease. *Neurosci. Lett.* 186 189–191. 10.1016/0304-3940(95)11297-A 7777193

[B85] Jones M. Peden A. H. Prowse C. V. Gröner A. Manson J. C. Turner M. L. (2007). In vitro amplification and detection of variant Creutzfeldt-Jakob disease PrPSc. *J. Pathol.* 213 21–26. 10.1002/path.2204 17614097

[B86] Kascsak R. J. Rubenstein R. Merz P. A. Tonna-DeMasi M. Fersko R. Carp R. I. (1987). Mouse polyclonal and monoclonal antibody to scrapie-associated fibril proteins. *J. Virol.* 61 3688–3693. 10.1128/jvi.61.12.3688-3693.1987 2446004 PMC255980

[B87] Kenney K. Brechtel C. Takahashi H. Kurohara K. Anderson P. Gibbs C. J. (2000). An enzyme-linked immunosorbent assay to quantify 14-3-3 proteins in the cerebrospinal fluid of suspected Creutzfeldt-Jakob disease patients. *Ann. Neurol.* 48 395–398. 10.1002/1531-8249(200009)48:3<395::AID-ANA18<3.0.CO;2-A 10976650

[B88] Kim C. Haldiman T. Cohen Y. Chen W. Blevins J. Sy M. S. (2011). Protease-sensitive conformers in broad spectrum of distinct prp sc structures in sporadic creutzfeldt-jakob disease are indicator of progression rate. *PLoS Pathog.* 7:e1002242. 10.1371/journal.ppat.1002242 21931554 PMC3169556

[B89] Kim C. Haldiman T. Surewicz K. Cohen Y. Chen W. Blevins J. (2012). Small protease sensitive oligomers of PrPSc in distinct human prions determine conversion rate of PrPC. *PLoS Pathog.* 8:e1002835. 10.1371/journal.ppat.1002835 22876179 PMC3410855

[B90] Kim C. Xiao X. Chen S. Haldiman T. Smirnovas V. Kofskey D. (2018). Artificial strain of human prions created in vitro. *Nat. Commun.* 9:2166. 10.1038/s41467-018-04584-z 29867164 PMC5986862

[B91] Kim M. O. Takada L. T. Wong K. Forner S. A. Geschwind M. D. (2018). Genetic PrP prion diseases. *Cold Spring Harb. Perspect. Biol.* 10:a033134. 10.1101/cshperspect.a033134 28778873 PMC5932589

[B92] Kobayashi A. Mizukoshi K. Iwasaki Y. Miyata H. Yoshida Y. Kitamoto T. (2011). Co-occurrence of types 1 and 2 PrPres in sporadic Creutzfeldt-Jakob disease MM1. *Am. J. Pathol.* 178 1309–1315. 10.1016/j.ajpath.2010.11.069 21356381 PMC3069892

[B93] Kraus A. Anson K. J. Raymond L. D. Martens C. Groveman B. R. Dorward D. W. (2015). Prion protein prolines 102 and 105 and the surrounding lysine cluster impede amyloid formation. *J. Biol. Chem.* 290 21510–21522. 10.1074/jbc.M115.665844 26175152 PMC4571877

[B94] Kraus A. Groveman B. R. Caughey B. (2013). Prions and the potential transmissibility of protein misfolding diseases. *Annu. Rev. Microbiol.* 67 543–564. 10.1146/annurev-micro-092412-155735 23808331 PMC4784231

[B95] Kruse N. Heslegrave A. Gupta V. Foiani M. Villar-Piqué A. Schmitz M. (2018). Interlaboratory validation of cerebrospinal fluid α-synuclein quantification in the diagnosis of sporadic Creutzfeldt-Jakob disease. *Alzheimer’s Dement.* 10 461–470. 10.1016/j.dadm.2018.06.005 30294658 PMC6171371

[B96] Kushnirov V. V. Dergalev A. A. Alexandrov A. I. (2020). Proteinase K resistant cores of prions and amyloids. *Prion* 14 11–19. 10.1080/19336896.2019.1704612 31876447 PMC6959286

[B97] Kwon G. T. Kwon M. S. (2019). Diagnostic challenge of rapidly progressing sporadic Creutzfeldt-Jakob disease. *BMJ Case Rep.* 12:e230535. 10.1136/bcr-2019-230535 31551319 PMC6768346

[B98] Lacroux C. Comoy E. Moudjou M. Perret-Liaudet A. Lugan S. Litaise C. (2014). Preclinical detection of variant CJD and BSE prions in blood. *PLoS Pathog.* 10:e1004202. 10.1371/journal.ppat.1004202 24945656 PMC4055790

[B99] Ladogana A. Sanchez-Juan P. Mitrová E. Green A. Cuadrado-Corrales N. Sánchez-Valle R. (2009). Cerebrospinal fluid biomarkers in human genetic transmissible spongiform encephalopathies. *J. Neurol.* 256 1620–1628. 10.1007/s00415-009-5163-x 19444528 PMC3085782

[B100] Lemstra A. W. Van Meegen M. T. Vreyling J. P. Meijerink P. H. S. Jansen G. H. Bulk S. (2000). 14-3-3 testing in diagnosing Creutzfeldt-Jakob disease: a prospective study in 112 patients. *Neurology* 55 514–516. 10.1212/WNL.55.4.514 10953182

[B101] Letourneau-Guillon L. Wada R. Kucharczyk W. (2012). Imaging of prion diseases. *J. Magn. Reson. Imaging* 35 998–1012. 10.1002/jmri.23504 22499277

[B102] Llewelyn C. A. Hewitt P. E. Knight R. S. G. Amar K. Cousens S. MacKenzie J. (2004). Possible transmission of variant Creutzfeldt-Jakob disease by blood transfusion. *Lancet* 363 417–421. 10.1016/S0140-6736(04)15486-X 14962520

[B103] Llorens F. Kruse N. Karch A. Schmitz M. Zafar S. Gotzmann N. (2018). Validation of α-Synuclein as a CSF biomarker for sporadic Creutzfeldt-Jakob disease. *Mol. Neurobiol.* 55 2249–2257. 10.1007/s12035-017-0479-5 28321768 PMC5840235

[B104] Llorens F. Kruse N. Schmitz M. Gotzmann N. Golanska E. Thüne K. (2017). Evaluation of α-synuclein as a novel cerebrospinal fluid biomarker in different forms of prion diseases. *Alzheimer’s Dement.* 13 710–719. 10.1016/j.jalz.2016.09.013 27870938

[B105] Llorens F. Rübsamen N. Hermann P. Schmitz M. Villar-Piqué A. Goebel S. (2020a). A prognostic model for overall survival in sporadic Creutzfeldt-Jakob disease. *Alzheimer’s Dement.* 16, 1438–1447. 10.1002/alz.12133 32614136

[B106] Llorens F. Villar-Piqué A. Hermann P. Schmitz M. Goebel S. Waniek K. (2020b). Cerebrospinal fluid non-phosphorylated tau in the differential diagnosis of Creutzfeldt–Jakob disease: a comparative prospective study with 14-3-3. *J. Neurol.* 267 543–550. 10.1007/s00415-019-09610-8 31701333

[B107] Llorens F. Villar-Piqué A. Schmitz M. Diaz-Lucena D. Wohlhage M. Hermann P. (2020c). Plasma total prion protein as a potential biomarker for neurodegenerative dementia: diagnostic accuracy in the spectrum of prion diseases. *Neuropathol. Appl. Neurobiol.* 46 240–254. 10.1111/nan.12573 31216593

[B108] Luk C. Jones S. Thomas C. Fox N. C. Tze H. (2016). Prospective diagnosis of sporadic CJD by the detection of abnormal PrP in patient urine. *JAMA Neurol.* 73 1454–1460. 10.1001/jamaneurol.2016.3733 27699415 PMC5701732

[B109] Lukan A. Vranac T. Èurin Šerbec V. (2013). TSE diagnostics: recent advances in immunoassaying prions. *Clin. Dev. Immunol.* 2013:360604. 10.1155/2013/360604 23970925 PMC3732588

[B110] Manix M. Kalakoti P. Henry M. Thakur J. Menger R. Guthikonda B. (2015). Creutzfeldt-Jakob disease: updated diagnostic criteria, treatment algorithm, and the utility of brain biopsy. *Neurosurg. Focus* 39:E2. 10.3171/2015.8.FOCUS15328 26646926

[B111] McGuire L. I. Peden A. H. Orrú C. D. Wilham J. M. Appleford N. E. Mallinson G. (2012). Real time quaking-induced conversion analysis of cerebrospinal fluid in sporadic Creutzfeldt-Jakob disease. *Ann. Neurol.* 72 278–285. 10.1002/ana.23589 22926858 PMC3458796

[B112] McGuire L. I. Poleggi A. Poggiolini I. Suardi S. Grznarova K. Shi S. (2016). Cerebrospinal fluid real-time quaking-induced conversion is a robust and reliable test for sporadic creutzfeldt-jakob disease: an international study. *Ann. Neurol.* 80 160–165. 10.1002/ana.24679 27130376 PMC4982084

[B113] Mente K. P. O’Donnell J. K. Jones S. E. Cohen M. L. Thompson N. R. Bizzi A. (2017). Fluorodeoxyglucose positron emission tomography (FDG-PET) correlation of histopathology and MRI in prion disease. *Alzheimer Dis. Assoc. Disord.* 31 1–7. 10.1097/WAD.0000000000000188 28121634 PMC5322151

[B114] Metrick M. A. do Carmo Ferreira N. Saijo E. Hughson A. G. Kraus A. Orrú C. (2019). Million-fold sensitivity enhancement in proteopathic seed amplification assays for biospecimens by Hofmeister ion comparisons. *Proc. Natl. Acad. Sci. U.S.A.* 116 23029–23039. 10.1073/pnas.1909322116 31641070 PMC6859373

[B115] Mielke M. M. Hagen C. E. Wennberg A. M. V. Airey D. C. Savica R. Knopman D. S. (2017). Association of plasma total tau level with cognitive decline and risk of mild cognitive impairment or dementia in the Mayo Clinic study on aging. *JAMA Neurol.* 74 1073–1080. 10.1001/jamaneurol.2017.1359 28692710 PMC5710182

[B116] Minikel E. V. Kuhn E. Cocco A. R. Vallabh S. M. Hartigan C. R. Reidenbach A. G. (2019). Domain-specific quantification of prion protein in cerebrospinal fluid by targeted mass spectrometry. *Mol. Cell. Proteomics* 18 2388–2400. 10.1074/mcp.RA119.001702 31558565 PMC6885701

[B117] Moda F. Gambetti P. Notari S. Concha-Marambio L. Catania M. Park K.-W. (2014). Prions in the urine of patients with variant Creutzfeldt–Jakob Disease. *N. Engl. J. Med.* 371 530–539. 10.1056/NEJMoa1404401 25099577 PMC4162740

[B118] Murray K. (2011). Creutzfeldt-jacob disease mimics, or how to sort out the subacute encephalopathy patient. *Pract. Neurol.* 11 19–28. 10.1136/pgmj.2010.235721rep 21239650

[B119] Nonno R. Di Bari M. A. Cardone F. Vaccari G. Fazzi P. Dell’Omo G. (2006). Efficient transmission and characterization of Creutzfeldt-Jakob disease strains in bank voles. *PLoS Pathog.* 2:e12. 10.1371/journal.ppat.0020012 16518470 PMC1383487

[B120] Orrú C. D. Bongianni M. Tonoli G. Ferrari S. Hughson A. G. Groveman B. R. (2014). A test for Creutzfeldt–Jakob Disease using nasal brushings. *N. Engl. J. Med.* 371 519–529. 10.1056/nejmoa1315200 25099576 PMC4186748

[B121] Orrú C. D. Groveman B. R. Hughson A. G. Zanusso G. Coulthart M. B. Caughey B. (2015a). Rapid and sensitive RT-QuIC detection of human creutzfeldt-jakob disease using cerebrospinal fluid. *mBio* 6:e02451-14. 10.1128/mBio.02451-14 25604790 PMC4313917

[B122] Orrú C. D. Groveman B. R. Raymond L. D. Hughson A. G. Nonno R. Zou W. (2015b). Bank vole prion protein as an apparently universal substrate for RT-QuIC-Based Detection and discrimination of prion strains. *PLoS Pathog.* 11:e1004983. 10.1371/journal.ppat.1004983 26086786 PMC4472236

[B123] Orrú C. D. Wilham J. M. Hughson A. G. Raymond L. D. McNally K. L. Bossers A. (2009). Human variant Creutzfeldt-Jakob disease and sheep scrapie PrPres detection using seeded conversion of recombinant prion protein. *Protein Eng. Des. Sel.* 22 515–521. 10.1093/protein/gzp031 19570812 PMC2719501

[B124] Orrú C. D. Wilham J. M. Raymond L. D. Kuhn F. Schroeder B. Raeber A. J. (2011). Prion disease blood test using immunoprecipitation and improved quaking-induced conversion. *mBio* 2:e00078-11. 10.1128/mBio.00078-11 21558432 PMC3101782

[B125] Orrú C. D. Yuan J. Appleby B. S. Li B. Li Y. Winner D. (2017). Prion seeding activity and infectivity in skin samples from patients with sporadic Creutzfeldt-Jakob disease. *Sci. Transl. Med.* 9:eaam7785. 10.1126/scitranslmed.aam7785 29167394 PMC5744860

[B126] Ortega-Cubero S. Luquín M. R. Domínguez I. Arbizu J. Pagola I. Carmona-Abellán M. M. (2013). Structural and functional neuroimaging in human prion diseases. *Neurología* 28 299–308. 10.1016/j.nrleng.2011.03.012 21621879

[B127] Oshita M. Yokoyama T. Takei Y. Takeuchi A. Ironside J. W. Kitamoto T. (2016). Efficient propagation of variant Creutzfeldt-Jakob disease prion protein using the cell-protein misfolding cyclic amplification technique with samples containing plasma and heparin. *Transfusion* 56 223–230. 10.1111/trf.13279 26347231

[B128] Otto M. Wiltfang J. Cepek L. Neumann M. Mollenhauer B. Steinacker P. (2002). Tau protein and 14-3-3 protein in the differential diagnosis of Creutzfeldt-Jakob disease. *Neurology* 58 192–197. 10.1212/WNL.58.2.192 11805244

[B129] Otto M. Wiltfang J. Schütz E. Zerr I. Otto A. Pfahlberg A. (1998). Diagnosis of Creutzfeldt-Jakob disease by measurement of S100 protein in serum: prospective case-control study. *Br. Med. J.* 316 577–582. 10.1136/bmj.316.7131.577 9518907 PMC28459

[B130] Otto M. Wiltfang J. Tumani H. Zerr I. Lantsch M. Kornhuber J. (1997). Elevated levels of tau-protein in cerebrospinal fluid of patients with Creutzfeldt-Jakob disease. *Neurosci. Lett.* 225 210–212. 10.1016/S0304-3940(97)00215-2 9147407

[B131] Parchi P. De Boni L. Saverioni D. Cohen M. L. Ferrer I. Gambetti P. (2012). Consensus classification of human prion disease histotypes allows reliable identification of molecular subtypes: an inter-rater study among surveillance centres in Europe and USA. *Acta Neuropathol.* 124 517–529. 10.1007/s00401-012-1002-8 22744790 PMC3725314

[B132] Parchi P. Giese A. Capellari S. Brown P. Schulz-Schaeffer W. Windl O. (1999). Classification of sporadic Creutzfeldt-Jakob disease based on molecular and phenotypic analysis of 300 subjects. *Ann. Neurol.* 46 224–233. 10.1002/1531-8249(199908)46:2<224::AID-ANA12<3.0.CO;2-W 10443888

[B133] Pastrana M. A. Sajnani G. Onisko B. Castilla J. Morales R. Soto C. (2006). Isolation and characterization of a proteinase K-sensitive PrPSc fraction. *Biochemistry* 45 15710–15717. 10.1021/bi0615442 17176093

[B134] Peden A. H. McGuire L. I. Appleford N. E. J. Mallinson G. Wilham J. M. Orrú C. D. (2012). Sensitive and specific detection of sporadic Creutzfeldt-Jakob disease brain prion protein using real-time quaking-induced conversion. *J. Gen. Virol.* 93 438–449. 10.1099/vir.0.033365-0 22031526 PMC3352348

[B135] Peggion C. Bertoli A. Sorgato M. C. (2017). Almost a century of prion protein(s): from pathology to physiology, and back to pathology. *Biochem. Biophys. Res. Commun.* 483 1148–1155. 10.1016/j.bbrc.2016.07.118 27581199

[B136] Peoc’h K. Schröder H. C. Laplanche J. L. Ramljak S. Müller W. E. G. (2001). Determination of 14-3-3 protein levels in cerebrospinal fluid from Creutzfeldt-Jakob patients by a highly sensitive capture assay. *Neurosci. Lett.* 301 167–170. 10.1016/S0304-3940(01)01619-6 11257424

[B137] Properzi F. Pocchiari M. (2013). Identification of misfolded proteins in body fluids for the diagnosis of prion diseases. *Int. J. Cell Biol.* 2013:839329. 10.1155/2013/839329 24027585 PMC3763259

[B138] Prusiner S. (1982). Novel proteinaceous infectious particles cause scrapie. *Science* 216 136–144. 10.1126/science.6801762 6801762

[B139] Prusiner S. B. (1991). Molecular biology of prion diseases. *Science* 252 1515–1522. 10.1126/science.1675487 1675487

[B140] Prusiner S. B. (1998). Prions. *Proc. Natl. Acad. Sci. U.S.A.* 95 13363–13383. 10.1073/pnas.95.23.13363 9811807 PMC33918

[B141] Prusiner S. B. Hsiao K. K. (1994). Human prion diseases. *Ann. Neurol.* 35 385–395. 10.1002/ana.410350404 8154865

[B142] Puoti G. Bizzi A. Forloni G. Safar J. G. Tagliavini F. Gambetti P. (2012). Sporadic human prion diseases: molecular insights and diagnosis. *Lancet Neurol.* 11 618–628. 10.1016/S1474-4422(12)70063-7 22710755

[B143] Raymond G. J. Race B. Orrú C. D. Raymond L. D. Bongianni M. Fiorini M. (2020). Transmission of CJD from nasal brushings but not spinal fluid or RT-QuIC product. *Ann. Clin. Transl. Neurol.* 7 932–944. 10.1002/acn3.51057 32538552 PMC7318090

[B144] Redaelli V. Bistaffa E. Zanusso G. Salzano G. Sacchetto L. Rossi M. (2017). Detection of prion seeding activity in the olfactory mucosa of patients with Fatal Familial Insomnia. *Sci. Rep.* 7 1–8. 10.1038/srep46269 28387370 PMC5384244

[B145] Renard D. Castelnovo G. Collombier L. Thouvenot E. Boudousq V. (2017). FDG-PET in Creutzfeldt-Jakob disease: analysis of clinical-PET correlation. *Prion* 11 440–453. 10.1080/19336896.2017.1387348 29099286 PMC5786355

[B146] Requena J. R. Wille H. (2017). The Structure of the Infectious Prion Protein and Its Propagation. *Prog. Mol. Biol. Transl. Sci.* 150 341–359. 10.1016/bs.pmbts.2017.06.009 28838667

[B147] Riek R. Hornemann S. Wider G. Billeter M. Glockshuber R. Wüthrich K. (1996). NMR structure of the mouse prion protein domain PrP(121–231). *Nature* 382 180–182. 10.1038/382180a0 8700211

[B148] Ritchie D. L. Ironside J. W. (2017). Neuropathology of Human Prion Diseases. *Prog. Mol. Biol. Transl. Sci.* 150 319–339. 10.1016/bs.pmbts.2017.06.011 28838666

[B149] Rossi G. Macchi G. Porro M. Giaccone G. Bugiani M. Scarpini E. (1998). Fatal familial insomnia: genetic, neuropathologic, and biochemical study of a patient from a new Italian kindred. *Neurology* 50 688–692. 10.1212/WNL.50.3.688 9521257

[B150] Rossi M. Baiardi S. Parchi P. (2019). Understanding prion strains: evidence from studies of the disease forms affecting humans. *Viruses* 11:309. 10.3390/v11040309 30934971 PMC6520670

[B151] Rudge P. Hyare H. Green A. Collinge J. Mead S. (2018). Imaging and CSF analyses effectively distinguish CJD from its mimics. *J. Neurol. Neurosurg. Psychiatry* 89 461–466. 10.1136/jnnp-2017-316853 29142140 PMC5909756

[B152] Saá P. Castilla J. Soto C. (2005). Cyclic amplification of protein misfolding and aggregation. *Methods Mol. Biol.* 2005 53–65. 10.1385/1-59259-874-9:053 15980595

[B153] Saá P. Castilla J. Soto C. (2006). Ultra-efficient replication of infectious prions by automated protein misfolding cyclic amplification. *J. Biol. Chem.* 281 35245–35252. 10.1074/jbc.M603964200 16982620

[B154] Saborio G. P. Permanne B. Soto C. (2001). Sensitive detection of pathological prion protein by cyclic amplification of protein misfolding. *Nature* 411 810–813. 10.1038/35081095 11459061

[B155] Safar J. Geschwind M. Deering C. Didorenko S. Sattavat M. Sanchez H. (2005). Diagnosis of human prion disease. *Proc. Natl. Acad. Sci. U.S.A.* 102 3501–3506. 10.1073/pnas.0409651102 15741275 PMC552933

[B156] Safar J. Wille H. Itri V. Groth D. Serban H. Torchia M. (1998). Eight prion strains have PrPSc molecules with different conformations. *Nat. Med.* 4 1157–1165. 10.1038/2654 9771749

[B157] Saijo E. Hughson A. G. Raymond G. J. Suzuki A. Horiuchi M. Caughey B. (2016). PrP Sc -specific antibody reveals c-terminal conformational differences between prion strains. *J. Virol.* 90 4905–4913. 10.1128/jvi.00088-16 26937029 PMC4859706

[B158] Sajnani G. Silva C. J. Ramos A. Pastrana M. A. Onisko B. C. Erickson M. L. (2012). PK-sensitive PrPSc is infectious and shares basic structural features with PK-resistant PrPSc. *PLoS Pathog.* 8:e1002547. 10.1371/journal.ppat.1002547 22396643 PMC3291653

[B159] Sanchez-Juan P. Green A. Ladogana A. Cuadrado-Corrales N. Sáanchez-Valle R. Mitrováa E. (2006). CSF tests in the differential diagnosis of Creutzfeldt-Jakob disease. *Neurology* 67 637–643. 10.1212/01.wnl.0000230159.67128.00 16924018

[B160] Sanchez-Juan P. Sánchez-Valle R. Green A. Ladogana A. Cuadrado-Corrales N. Mitrová E. (2007). Influence of timing on CSF tests value for Creutzfeldt-Jakob disease diagnosis. *J. Neurol.* 254 901–906. 10.1007/s00415-006-0472-9 17385081 PMC2779401

[B161] Sano K. Satoh K. Atarashi R. Takashima H. Iwasaki Y. Yoshida M. (2013). Early detection of abnormal prion protein in genetic human prion diseases now possible using real-time QUIC assay. *PLoS One* 8:e54915. 10.1371/journal.pone.0054915 23372790 PMC3556051

[B162] Savard M. Irani S. R. Guillemette A. Gosselin-Lefebvre S. Geschwind M. Jansen G. H. (2016). Creutzfeldt-Jakob disease-like periodic sharp wave complexes in voltage-gated potassium channel-complex antibodies encephalitis: a case report. *J. Clin. Neurophysiol.* 33 e1–e4. 10.1097/WNP.0000000000000171 26375660 PMC4740210

[B163] Scheckel C. Aguzzi A. (2018). Prions, prionoids and protein misfolding disorders. *Nat. Rev. Genet.* 19 405–418. 10.1038/s41576-018-0011-4 29713012

[B164] Schmitz M. Ebert E. Stoeck K. Karch A. Collins S. Calero M. (2016). Validation of 14-3-3 protein as a marker in sporadic Creutzfeldt-Jakob Disease Diagnostic. *Mol. Neurobiol.* 53 2189–2199. 10.1007/s12035-015-9167-5 25947081

[B165] Schmitz M. Villar-Piqué A. Llorens F. Gmitterová K. Hermann P. Varges D. (2019). Cerebrospinal fluid total and phosphorylated α-synuclein in patients with Creutzfeldt–Jakob Disease and synucleinopathy. *Mol. Neurobiol.* 56 3476–3483. 10.1007/s12035-018-1313-4 30136097

[B166] Scott M. R. Peretz D. Nguyen H.-O. B. DeArmond S. J. Prusiner S. B. (2005). Transmission barriers for bovine, ovine, and human prions in transgenic mice. *J. Virol.* 79 5259–5271. 10.1128/jvi.79.9.5259-5271.2005 15827140 PMC1082721

[B167] Sharma A. Bruce K. L. Chen B. Gyoneva S. Behrens S. H. Bommarius A. S. (2016). Contributions of the prion protein sequence, strain, and environment to the species barrier. *J. Biol. Chem.* 291 1277–1288. 10.1074/jbc.M115.684100 26565023 PMC4714215

[B168] Smirnovas V. Baron G. S. Offerdahl D. K. Raymond G. J. Caughey B. Surewicz W. K. (2011). Structural organization of brain-derived mammalian prions examined by hydrogen-deuterium exchange. *Nat. Struct. Mol. Biol.* 18 504–506. 10.1038/nsmb.2035 21441913 PMC3379881

[B169] Soto C. (2004). Diagnosing prion diseases: needs, challenges and hopes. *Nat. Rev. Microbiol.* 2 809–819. 10.1038/nrmicro1003 15378045

[B170] Soto C. Anderes L. Suardi S. Cardone F. Castilla J. Frossard M. J. (2005). Pre-symptomatic detection of prions by cyclic amplification of protein misfolding. *FEBS Lett.* 579 638–642. 10.1016/j.febslet.2004.12.035 15670821

[B171] Soto C. Satani N. (2011). The intricate mechanisms of neurodegeneration in prion diseases. *Trends Mol. Med.* 17 14–24. 10.1016/j.molmed.2010.09.001 20889378 PMC3056171

[B172] Spagnolli G. Rigoli M. Orioli S. Sevillano A. M. Faccioli P. Wille H. (2019). Full atomistic model of prion structure and conversion. *PLoS Pathog.* 15:e1007864. 10.1371/journal.ppat.1007864 31295325 PMC6622554

[B173] Staffaroni A. M. Kramer A. O. Casey M. Kang H. Rojas J. C. Orrú C. D. (2019). Association of blood and cerebrospinal fluid tau level and other biomarkers with survival time in sporadic Creutzfeldt-Jakob Disease. *JAMA Neurol.* 76 969–977. 10.1001/jamaneurol.2019.1071 31058916 PMC6503575

[B174] Steinacker P. Blennow K. Halbgebauer S. Shi S. Ruf V. Oeckl P. (2016). Neurofilaments in blood and CSF for diagnosis and prediction of onset in Creutzfeldt-Jakob disease. *Sci. Rep.* 6:38737. 10.1038/srep38737 27929120 PMC5144074

[B175] Stoeck K. Sanchez-Juan P. Gawinecka J. Green A. Ladogana A. Pocchiari M. (2012). Cerebrospinal fluid biomarker supported diagnosis of Creutzfeldt-Jakob disease and rapid dementias: a longitudinal multicentre study over 10 years. *Brain* 135 3051–3061. 10.1093/brain/aws238 23012332 PMC3470713

[B176] Tee B. L. Longoria Ibarrola E. M. Geschwind M. D. (2018). Prion Diseases. *Neurol. Clin.* 36 865–897. 10.1016/j.ncl.2018.07.005 30366560 PMC13193203

[B177] Tesar A. Matej R. Kukal J. Johanidesova S. Rektorova I. Vyhnalek M. (2019). Clinical variability in P102L Gerstmann–Sträussler–Scheinker syndrome. *Ann. Neurol.* 86 643–652. 10.1002/ana.25579 31397917

[B178] The 1000 Genomes Project Consortium (2012). An integrated map of genetic variation from 1,092 human genomes. *Nature* 491 56–65. 10.1038/nature11632 23128226 PMC3498066

[B179] Thomas J. G. Chenoweth C. E. Sullivan S. E. (2013). Iatrogenic Creutzfeldt-Jakob disease via surgical instruments. *J. Clin. Neurosci.* 20 1207–1212. 10.1016/j.jocn.2013.01.007 23896549

[B180] Thompson A. Anastasiadis P. Druyeh R. Whitworth I. Nayak A. Nihat A. (2020). Evaluation of plasma tau and neurofilament light chain biomarkers in a 12-year clinical cohort of human prion diseases. *medRxiv* [Preprint]. 10.1101/2020.07.27.20157594 PMC8758487 33674752

[B181] Thompson A. G. B. Luk C. Heslegrave A. J. Zetterberg H. Mead S. H. Collinge J. (2018). Neurofilament light chain and tau concentrations are markedly increased in the serum of patients with sporadic Creutzfeldt-Jakob disease, and tau correlates with rate of disease progression. *J. Neurol. Neurosurg. Psychiatry* 89 955–961. 10.1136/jnnp-2017-317793 29487167 PMC6109239

[B182] Turner M. L. Ironside J. W. (1998). New-variant Creutzfeldt-Jakob disease: the risk of transmission by blood transfusion. *Blood Rev.* 12 255–268. 10.1016/S0268-960X(98)90007-8 9950096

[B183] Uttley L. Carroll C. Wong R. Hilton D. A. Stevenson M. (2020). Creutzfeldt-Jakob disease: a systematic review of global incidence, prevalence, infectivity, and incubation. *Lancet Infect. Dis.* 20 e2–e10. 10.1016/S1473-3099(19)30615-2 31876504

[B184] Vallabh S. M. Minikel E. V. Williams V. J. Carlyle B. C. McManus A. J. Wennick C. D. (2020). Cerebrospinal fluid and plasma biomarkers in individuals at risk for genetic prion disease. *BMC Med.* 18:140. 10.1186/s12916-020-01608-8 32552681 PMC7302371

[B185] Vallabh S. M. Nobuhara C. K. Llorens F. Zerr I. Parchi P. Capellari S. (2019). Prion protein quantification in human cerebrospinal fluid as a tool for prion disease drug development. *Proc. Natl. Acad. Sci. U.S.A.* 116 7793–7798. 10.1073/pnas.1901947116 30936307 PMC6475435

[B186] Van Everbroeck B. Quoilin S. Boons J. Martin J. J. Cras P. (2003). A prospective study of CSF markers in 250 patients with possible Creutzfeldt-Jakob disease. *J. Neurol. Neurosurg. Psychiatry* 74 1210–1214. 10.1136/jnnp.74.9.1210 12933920 PMC1738637

[B187] Villar-Piqué A. Schmitz M. Lachmann I. Karch A. Calero O. Stehmann C. (2019). Cerebrospinal fluid total prion protein in the spectrum of prion diseases. *Mol. Neurobiol.* 56 2811–2821. 10.1007/s12035-018-1251-1 30062673

[B188] Wadsworth J. Hill A. Beck J. Collinge J. (2003). Molecular and clinical classification of human prion disease. *Br. Med. Bull.* 66 241–254. 10.1093/bmb/dg66.241 14522862

[B189] Wang Z. Manca M. Foutz A. Camacho M. V. Raymond G. J. Race B. (2019). Early preclinical detection of prions in the skin of prion-infected animals. *Nat. Commun.* 10:247. 10.1038/s41467-018-08130-9 30651538 PMC6335425

[B190] Wasmer C. Lange A. Van Melckebeke H. Siemer A. B. Riek R. Meier B. H. (2008). Amyloid fibrils of the HET-s(218-289) prion form a β solenoid with a triangular hydrophobic core. *Science* 319 1523–1526. 10.1126/science.1151839 18339938

[B191] Watts J. C. Giles K. Patel S. Oehler A. DeArmond S. J. Prusiner S. B. (2014). Evidence That Bank Vole PrP Is a Universal Acceptor for Prions. *PLoS Pathog.* 10:e1003990. 10.1371/journal.ppat.1003990 24699458 PMC3974871

[B192] Wells G. A. Scott A. C. Johnson C. T. Gunning R. F. Hancock R. D. Jeffrey M. (1987). A novel progressive spongiform encephalopathy in cattle. *Vet. Rec.* 121 419–420. 10.1136/vr.121.18.419 3424605

[B193] Weydt P. Oeckl P. Huss A. Müller K. Volk A. E. Kuhle J. (2016). Neurofilament levels as biomarkers in asymptomatic and symptomatic familial amyotrophic lateral sclerosis. *Ann. Neurol.* 79 152–158. 10.1002/ana.24552 26528863

[B194] Whitechurch B. C. Welton J. M. Collins S. J. Lawson V. A. (2017). Clinical Aspects of Alzheimer’s Disease Chapter 13 Prion Diseases. *Adv. Neurobiol.* 15 335–364. 10.1007/978-3-319-57193-5 28674988

[B195] Wieser H. G. Schindler K. Zumsteg D. (2006). EEG in Creutzfeldt-Jakob disease. *Clin. Neurophysiol.* 117 935–951. 10.1016/j.clinph.2005.12.007 16442343

[B196] Wilham J. M. Orrú C. D. Bessen R. A. Atarashi R. Sano K. Race B. (2010). Rapid end-point quantitation of prion seeding activity with sensitivity comparable to bioassays. *PLoS Pathog.* 6:e1001217. 10.1371/journal.ppat.1001217 21152012 PMC2996325

[B197] Will R. Ironside J. Zeidler M. Estibeiro K. Cousens S. Smith P. (1996). A new variant of Creutzfeldt-Jakob disease in the UK. *Lancet* 347 921–925. 10.1016/S0140-6736(96)91412-9 8598754

[B198] Will R. G. (2003). Acquired prion disease: iatrogenic CJD, variant CJD, kuru. *Br. Med. Bull.* 66 255–265. 10.1093/bmb/66.1.255 14522863

[B199] Wu L. Y. Zhan S. Q. Huang Z. Y. Zhang B. Wang T. Liu C. F. (2018). Expert consensus on clinical diagnostic criteria for fatal familial insomnia. *Chin. Med. J. Engl.* 131 1613–1617. 10.4103/0366-6999.235115 29941716 PMC6032681

[B200] Zahn R. Liu A. Lührs T. Riek R. von Schroetter C. López García F. (2000). NMR solution structure of the human prion protein. *Proc. Natl. Acad. Sci. U.S.A.* 97 145–150. 10.1073/pnas.97.1.145 10618385 PMC26630

[B201] Zanusso G. Monaco S. Pocchiari M. Caughey B. (2016). Advanced tests for early and accurate diagnosis of Creutzfeldt-Jakob disease. *Nat. Rev. Neurol.* 12 325–333. 10.1038/nrneurol.2016.65 27174240

[B202] Zerr I. Bodemer M. Gefeller O. Otto M. Poser S. Wiltfang J. (1998). Detection of 14-3-3 protein in the cerebrospinal fluid supports the diagnosis of Creutzfeldt-Jakob disease. *Ann. Neurol.* 43 32–40. 10.1002/ana.410430109 9450766

[B203] Zerr I. Bodemer M. Räcker S. Grosche S. Poser S. Weber T. (1995). Cerebrospinal fluid concentration of neuron-specific enolase in diagnosis of Creutzfeldt-Jakob disease. *Lancet* 345 1609–1610. 10.1016/S0140-6736(95)90118-3 7783539

[B204] Zerr I. Kallenberg K. Summers D. M. Romero C. Taratuto A. Heinemann U. (2009). Updated clinical diagnostic criteria for sporadic Creutzfeldt-Jakob disease. *Brain* 132 2659–2668. 10.1093/brain/awp191 19773352 PMC2759336

[B205] Zerr I. Pocchiari M. Collins S. Brandel J. P. De Pedro Cuesta J. Knight R. S. G. (2000a). Analysis of EEG and CSF 14-3-3 proteins as aids to the diagnosis of Creutzfeldt-Jakob disease. *Neurology* 55 811–815. 10.1212/WNL.55.6.811 10994001

[B206] Zerr I. Schulz-Schaeffer W. J. Giese A. Bodemer M. Schröter A. Henkel K. (2000b). Current clinical diagnosis in Creutzfeldt-Jakob disease: identification of uncommon variants. *Ann. Neurol.* 48 323–329. 10.1002/1531-8249(200009)48:3<323::AID-ANA6<3.0.CO;2-5 10976638

[B207] Zou W. Q. Capellari S. Parchi P. Sy M. S. Gambetti P. Chen S. G. (2003). Identification of novel proteinase k-resistant c-terminal fragments of PrP in Creutzfeldt-Jakob Disease. *J. Biol. Chem.* 278 40429–40436. 10.1074/jbc.M308550200 12917418

